# RRM2‐targeted nanocarrier enhances radiofrequency ablation efficacy in hepatocellular carcinoma through ferroptosis amplification and immune remodeling

**DOI:** 10.1002/imt2.70067

**Published:** 2025-08-06

**Authors:** Weiliang Hou, Weifeng Hong, Songhua Cai, Dandan Guo, Zhiping Yan, Jinyu Zhu, Yang Shen, Juncheng Wan, Xudong Qu, Wen Zhang, Runkang Zhao, Zhao Xie, Zhongji Chen, Tong Jiang, Yaling Lin, Wenlong Jia, Ling Wang, Zhao Huang, Xuexin Li, Bufu Tang

**Affiliations:** ^1^ Department of Gastroenterology, Shanghai Institute of Pancreatic Diseases, National Key Laboratory of Immunity and Inflammation, Changhai Clinical Research Unit, Changhai Hospital Naval Medical University Shanghai China; ^2^ Department of Radiation Oncology, Zhejiang Cancer Hospital; Hangzhou Institute of Medicine (HIM) Chinese Academy of Sciences Hangzhou China; ^3^ Department of Thoracic Surgery, National Cancer Center/National Clinical Research Center for Cancer/Cancer Hospital & Shenzhen Hospital Chinese Academy of Medical Sciences and Peking Union Medical College Shenzhen China; ^4^ First Affiliated Hospital Dalian Medical University Dalian China; ^5^ Department of Interventional Radiology, Zhongshan Hospital, Shanghai Institute of Medical Imaging, Shanghai Institution of Medical Imaging, Shanghai, National Clinical Research Center of Interventional Medicine Fudan University Shanghai China; ^6^ Key Laboratory of Carcinogenesis and Translational Research (Ministry of Education/Beijing), Department of Nuclear Medicine, Peking University Cancer Hospital & Institute Peking University Beijing China; ^7^ Department of Radiation Oncology Zhongshan Hospital Affiliated to Fudan University Shanghai China; ^8^ Hepatic Surgery Center, Tongji Hospital, Tongji Medical College Huazhong University of Science and Technology Wuhan China; ^9^ Department of General Surgery, The Fourth Affiliated Hospital China Medical University Shenyang China; ^10^ Department of Physiology and Pharmacology Karolinska Institute Solna Sweden

**Keywords:** ferroptosis, hepatocellular carcinoma, nanocarrier, radiofrequency ablation, ribonucleotide reductase M2

## Abstract

Hepatocellular carcinoma (HCC) is associated with high mortality rates despite the widespread application of radiofrequency ablation (RFA), which has limited therapeutic efficacy as a monotherapy. This study investigated ribonucleotide reductase M2 (*RRM2*) upregulation in post‐RFA HCC tissues and developed a targeted nanoco‐delivery system (red blood cell membrane/cRGD‐modified pH‐sensitive liposomes [sS@RBCM/cRGD‐phLips]) to increase RFA efficacy through specific *RRM2* knockout. *RRM2* knockout synergistically amplified RFA‐induced tumor cell death by promoting ferroptosis and immunogenic cell death. Mechanistically, *RRM2* knockout upregulated the STAT1–IRF1–ACSL4 axis, which potentiated lipid peroxidation and ferroptosis. Furthermore, the nanocarrier system enhanced dendritic cell maturation and cytotoxic T cell infiltration, thereby remodeling the tumor immune microenvironment. In vivo experiments revealed that the combination of RFA and RRM2‐targeted nanoparticles significantly suppressed tumor growth and prolonged survival in HCC‐bearing mice with minimal systemic toxicity. Notably, the dual‐loaded nanoparticles also enhanced the efficacy of anti‐programmed cell death protein 1 therapy, suggesting a promising combinatorial approach for HCC treatment. This study presents a novel therapeutic strategy that integrates *RRM2*‐targeted gene editing with RFA, offering a robust and synergistic approach for improving HCC outcomes.

## Highlights

- Ribonucleotide reductase M2 (*RRM2*)‐targeted nanocarriers synergistically enhance radiofrequency ablation (RFA) efficacy in hepatocellular carcinoma (HCC) by amplifying ferroptosis.
- Dual‐loaded red blood cell membrane/cRGD‐modified pH‐sensitive liposomes (sS@RBCM/cRGD‐phLips)‐phLips nanoplatform activates the STAT1‐IRF1‐ACSL4 pathway to promote ferroptosis.
- *RRM2* knockout remodels tumor immune microenvironment by enhancing DC maturation and CD8+ T cell infiltration.
- Combined therapy with anti‐PDL1 significantly improves tumor suppression and prolongs survival in HCC models.

## Graphical abstract

This study proposes an innovative therapeutic approach by combining RRM2 gene knockout with a nanocarrier system, significantly enhancing the efficacy of radiofrequency ablation for hepatocellular carcinoma. The study developed a tumor microenvironment‐responsive nanosystem that achieves synergistic effects through SPIO‐mediated and RRM2 knockout‐induced ferroptosis, along with precise targeting of tumor microenvironment regulation.

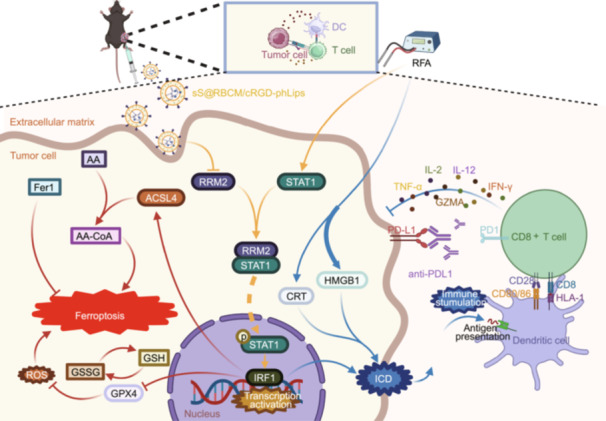

## INTRODUCTION

Hepatocellular carcinoma (HCC) is a major global health burden characterized by escalating incidence and mortality rates that pose substantial challenges to public health systems worldwide. HCC pathogenesis and progression are associated with chronic liver injury, which is characterized by complex interactions within the tumor microenvironment (TME) and dysregulated inflammatory cascades. Despite recent advances in therapeutic strategies and diagnostic modalities, monotherapeutic approaches remain inadequate for addressing the heterogeneity and recurrence propensity of HCC. Therefore, developing integrated multimodal therapeutic strategies and elucidating the underlying molecular mechanisms are imperative for optimizing HCC treatment outcomes.

Magnetic resonance imaging (MRI) has emerged as a pivotal tool for diagnosing HCC [1]. Superparamagnetic iron oxide (SPIO) is a valuable contrast agent used during MRI to diagnose HCC, facilitating in vivo cell labeling and tracking [2]. SPIO exhibits superior biocompatibility, enhanced cell and tumor tissue targeting, and improves the diagnostic accuracy of MRI [3]. Radiofrequency ablation (RFA) is a highly effective therapeutic modality for HCC, demonstrating substantial improvements in patient survival. Compared with alternative treatment modalities, RFA offers enhanced safety, reduced invasiveness, and accelerated patient recovery [4]. However, despite these advantages, the efficacy of RFA is often limited by incomplete ablation and tumor recurrence, necessitating the development of combinatorial therapeutic strategies.

Unlike the conventional apoptotic and necrotic pathways, ferroptosis orchestrates cell death through iron‐dependent lipid peroxidation cascades, demonstrating remarkable therapeutic potential in diverse malignancies. The TME, which encompasses blood vessels, immune cells, fibroblasts, signaling molecules, and the extracellular matrix, is a crucial determinant of HCC initiation and progression. The intricate interplay between the TME and ferroptosis offers compelling opportunities for the development of novel therapeutic strategies for HCC [5, 6]. Ferroptosis, a recently characterized form of regulated cell death, has demonstrated considerable therapeutic potential in cancer treatment [7, 8]. Furthermore, TME‐responsive nanoparticle codelivery systems can notably augment the efficacy of cancer treatment [9]. Therefore, modulating the immune microenvironment is a promising therapeutic strategy for the future management of HCC.

Ribonucleotide reductase M2 (*RRM2*) plays a pivotal role in maintaining DNA biosynthesis, repair, and replication. Moreover, *RRM2* has been implicated in tumor immunity [10] and has been extensively studied in lung, prostate, and kidney cancer [11, 12]. *RRM2* is frequently overexpressed in HCC, leading to the knockout of ferroptosis by modulating glutathione (GSH) levels [13]. Therefore, the induction of ferroptosis through *RRM2* knockout is a promising approach for cancer therapy. Furthermore, the modulation of *RRM2* enhances the radiosensitivity of neuroendocrine tumors [14]. However, the precise role of *RRM2* in modulating RFA‐induced HCC knockout and TME alterations, as well as its specific regulatory mechanisms, remains unclear.

The emergence of sophisticated gene‐editing technologies, particularly the clustered, regularly interspaced short palindromic repeats/CRISPR‐associated protein 9 (CRISPR/Cas9) system, has provided promising therapeutic approaches for cancer treatment [15]. The high‐efficiency gene knockout capability of CRISPR/Cas9 is instrumental in genetic analysis, and the sgRNA sequence serves as a regulatory element in CRISPR/Cas9‐mediated gene editing [16]. Targeted *RRM2* knockout via the CRISPR/Cas9 system, which suppresses gene expression and attenuates tumor cell proliferation and DNA repair capacity, offers a potential avenue for in vivo gene reediting as a therapeutic strategy for cancer [17]. The synergistic approach of *RRM2* targeting combined with nanoparticle‐based delivery systems holds promise as a valuable therapeutic modality for future cancer treatments.

Red blood cell membranes (RBCMs), derived from autologous red blood cells, exhibit excellent biocompatibility and safety. The immune evasion capability of the outer shell of the nanocarrier is markedly enhanced, thereby inhibiting macrophage phagocytosis and prolonging the circulation time of the nanocarrier in vivo [18, 19]. Additionally, pH‐sensitive liposomes (PhLips) can precisely release drugs under the acidic conditions of the TME, thus achieving tumor‐specific targeting and controlled drug release. Cyclic arginine‐glycine‐aspartic acid (cRGD) peptide, which can significantly increase the concentration of drugs at the tumor site, is used to further enhance the targeting ability of the nanocarrier to tumor cells, thereby improving therapeutic efficacy [20]. This multifunctional nanocarrier design enhances drug targeting and controlled release capabilities and provides an efficient and safe delivery platform for tumor treatment.

This study presents an innovative therapeutic paradigm to overcome the inherent limitations of RFA monotherapy. Enhanced therapeutic efficacy for HCC was achieved by integrating *RRM2* knockout and an advanced nanocarrier system. Central to this approach is the development of a TME‐responsive nanosystem (*sgRRM2*/SPIO@RBCM/cRGD‐phLips) that synergistically combined SPIO‐mediated MRI contrast enhancement, *RRM2* knockout‐induced ferroptosis, and precision‐targeted TME modulation. Comprehensive in vivo and in vitro investigations systematically elucidated the enhanced therapeutic efficacy of *RRM2* interference in combination with RFA and unveiled the molecular mechanisms underlying *RRM2*‐mediated ferroptosis and TME regulation.

## RESULTS

### *RRM2* is highly expressed in RFA‐treated HCC tissue, which is closely associated with poor prognosis in HCC

**FIGURE 1:**
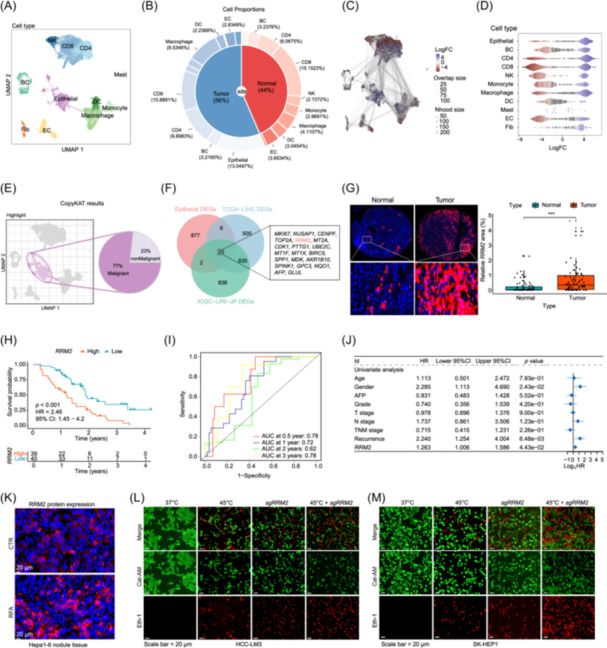
Ribonucleotide reductase *M2* (*RRM2*) is highly expressed in hepatocellular carcinoma (HCC) tissue after radiofrequency ablation (RFA) treatment, which is associated with poor prognosis in patients with HCC. (A) Uniform Manifold Approximation and Projection (UMAP) plot showing cell type annotations based on identified characteristic genes. The identified cell types included epithelial cell, B cell, CD4, CD8, NK cell, monocyte, macrophage, dendritic cell (DC), mast cell, endothelial cell (EC), and fibroblast. (B) Pie plot comparing the cellular composition of normal and tumor tissues. (C, D) Milo algorithm visualization of differential abundance testing on a k‐nearest neighbor graph and differential abundance analysis of each cell between normal and HCC tissues. (E) The CopyKAT algorithm distinguishes malignant from nonmalignant epithelial cells in tumor tissues. (F) Venn diagram showing the intersection of differentially expressed genes (DEGs) at the single‐cell and tissue levels, identifying 20 potential key molecules in HCC development. (G) Comparative analysis of *RRM2* expression levels in normal liver and HCC tissues. (H) Kaplan–Meier survival analysis of patients with HCC stratified by *RRM2* immunohistochemistry scores. (I) Receiver operating characteristic curve analysis of *RRM2* expression levels, demonstrating the prognostic value of the model for HCC. (J) Forest plot identifying *RRM2* as an independent risk factor for HCC. (K) Comparative analysis of RRM2 expression in control (CTR) and RFA HCC tissues. (L, M) Viability assessment of HCC‐LM3 (L) and SK‐HEP‐1 (M) cells using calcein AM (green) and ethidium homodimer‐1 (red) dual staining (*n* = 4). Scale bar = 20 μm.

Through single‐cell RNA sequencing analysis, we observed significant transcriptomic differences between tumor and normal tissue cells (Figure S1A,B). scRNA‐seq datasets from three independent cohorts were integrated, including previously published research [21], GSE242889 [22], and GSE149614 [23], totaling 34 samples (13 normal and 21 HCC samples (Figure S1C). Batch effect assessment demonstrated that the Harmony algorithm [24] significantly outperformed the PCA method in correcting for sample site, data set source, and donor differences (*p* < 0.001) (Figure S1D). Uniform Manifold Approximation and Projection analysis was used to eliminate pre‐existing batch differences stemming from donors, tissue types, and datasets. Finally, the gene expression profiles of 91,746 cells were retained (Figure S2A). To elucidate the HCC TME, a clustering analysis was performed on all cells and the corresponding clusters were annotated as epithelial cell, B cell, CD4+ T cell, CD8+ T cell, natural killer cell, monocyte, macrophage, dendritic cells (DC), mast cell, endothelial cell (EC), and fibroblast, based on the identified characteristic genes (Figures 1A and S1E). Comparative analysis of the cellular compositions from different tissue sources revealed significantly higher proportions of epithelial cells and macrophages in tumor tissues than in normal tissues (Figures 1B and S2B). The Milo algorithm [25] was used to assign single cells to partially overlapping neighborhoods on a k‐nearest neighbor graph for differential abundance testing of cellular components (Figure 1C). Consistent with Figure 1B, a significant increase in the proportion of epithelial cells in HCC tissues was observed compared to that in normal tissues (Figure 1D), which was potentially attributable to the proliferation of malignant cells in tumor samples. The CopyKAT algorithm [26], using epithelial cells from normal tissues as a reference, was applied to estimate gene copy numbers at an average genomic resolution from high‐throughput scRNA‐seq data of epithelial cells in tumor tissues. This approach enabled the distinction of malignant cells within the epithelial cell population of the tumor tissues (Figure 1E). Differential analysis at the single‐cell level was conducted between malignant and nonmalignant cells to further identify the key targets associated with HCC development, and a set of genes highly expressed in malignant cells was identified. Concurrently, tissue‐level differential analysis (HCC vs. normal) was performed using RNA‐seq data from the TCGA‐LIHC and ICGC‐LIRI‐JP cohorts. Integration of differentially expressed genes (DEGs) in a Venn diagram at both the cellular and tissue levels revealed 20 intersecting genes that were considered as potential key molecules influencing HCC development (Figure 1F). This multilevel, multidimensional analytical approach provides a comprehensive perspective, facilitating a deeper understanding of the molecular mechanisms underlying HCC and the identification of potential therapeutic targets.

Clinical samples from patients with HCC were collected for analysis, and normal liver tissue adjacent to the HCC lesions was used as a control. *RRM2* expression levels in HCC tissues were significantly higher than those in the adjacent normal liver tissues (Figure 1G). Patients with high *RRM2* expression exhibited significantly shorter survival times (Figures 1H and S2C–E). Receiver operating characteristic (curve analysis of *RRM2* as a prognostic marker for HCC revealed area under the curve values of 0.72, 0.62, and 0.78 at 1, 3, and 5 years, respectively (Figure 1I). Univariate analysis demonstrated that *RRM2* expression was a significant risk factor for poor prognosis in HCC (Figure 1J). Furthermore, RRM2 expression was upregulated after RFA treatment (Figure 1K). Western blot analysis was performed to detect RRM2 knockout; RRM2 protein expression was significantly decreased or almost eliminated in both the SK‐HEP1 and HCC‐LM3 cell lines (Figure S2F). Existing studies have found that moderate hyperthermic conditions of approximately 45°C can induce cellular DNA damage response, oxidative stress, and activation of various stress signaling pathways, while preserving sufficient cell viability to investigate their molecular response mechanisms. In line with this established approach [27], SK‐HEP1 and HCC‐LM3 HCC cells were heated in a 45°C water bath for 10 min to simulate the thermal ablation of in vitro RFA treatment. Both heat treatment and *RRM2* knockout significantly induced cell death (Figure 1L,M).

### Engineered lipid nanoparticles provide effective and stable delivery of *sgRRM2*, optimizing tumor targeting

**FIGURE 2:**
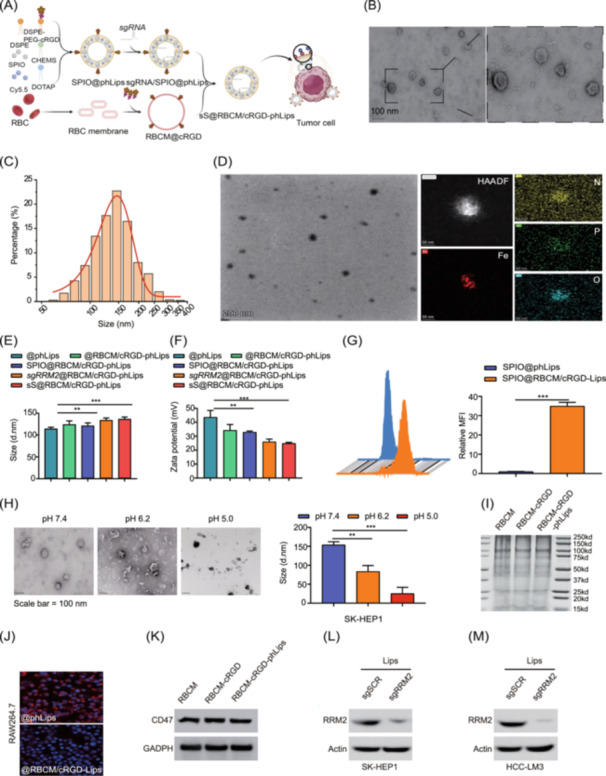
Engineered lipid nanoparticles (LNPs) effectively deliver *sgRRM2* with enhanced tumor‐targeting capabilities. (A) Schematic representation of the modified LNP structure, including dual‐targeting red blood cell membrane/cRGD‐modified pH‐sensitive liposomes (RBCM/cRGD‐phLips). (B) Representative transmission electron microscopy (TEM) micrographs showing the spherical morphology and uniform structure of the modified LNPs. (C) Dynamic light scattering analysis illustrating the size distribution of LNPs and confirming their nanoscale dimensions. (D) A tumor microenvironment (TME) plot depicting the particle size and dispersity of the LNP‐sgRNA formulations. (E) A statistical histogram comparing the sizes of the five LNP formulations showing a consistent size distribution. (F) Zeta potential measurements of the five LNP formulations, highlighting the differences in surface charge. (G) Nanoparticle flow cytometry histogram comparing the cellular uptake or binding of SPIO@phLips and SPIO@RBCM/cRGD‐Lips. (H) Analysis of the protein activity and stability of the nanocarriers under different pH conditions (7.4, 6.2, and 5.0). (I) Coomassie blue staining showing the protein composition in RBCM, RBCM‐cRGD conjugates, and dual‐targeting RBCM/cRGD‐phLips nanoparticles, confirming the successful incorporation of targeting ligands. (J) Representative immunofluorescence (IF) micrographs illustrating reduced Cy5.5‐labeled LNP uptake by macrophages, indicating immune evasion. (K) Western blot analysis of CD47 and GAPDH expression in RBCM, RBCM‐cRGDs, and RBCM/cRGD‐phLips showing the retention of CD47 as a marker of immune evasion. (L, M) Western blot analysis of RRM2 and actin expression in SK‐HEP1 (L) and HCC‐LM3 (M) cells after treatment with LNP‐encapsulated sgRRM2, demonstrating the efficient delivery and RRM2 knockout. Scale bar = 20 μm.

The modified nanoparticles (@RBCM/cRGD‐phLips) (Figure 2A) and transmission electron microscopy (TEM) analysis revealed the morphology of @RBCM/cRGD‐phLips (Figure 2B). The lipid nanoparticles (LNPs) displayed a hydrodynamic diameter of 136 ± 28 nm (Figure 2C). Gel electrophoresis analysis demonstrated the complete incorporation of sgRNA into the LNPs at a weight ratio of 1:4 (Figure S3A). TEM imaging further confirmed the presence of iron on the surface of the nanoparticle (Figure 2D). Zeta potential measurements and particle size distributions of the five engineered nanoparticle types are shown in Figure 2E,F (@phLips: 110 ± 5 nm, @RBCM/cRGD‐phLips: 120 ± 10 nm, SPIO@RBCM/cRGD‐phLips: 125 ± 8 nm, *sgRRM2*@RBCM/cRGD‐phLips: 140 ± 5 nm, sS@RBCM/cRGD‐phLips: 142 ± 7 nm, (@phLips: 43 ± 5 mV, @RBCM/cRGD‐phLips: 35 ± 4 mV, SPIO@RBCM/cRGD‐phLips: 33 ± 3 mV, *sgRRM2*@RBCM/cRGD‐phLips: 25 ± 2 mV, sS@RBCM/cRGD‐phLips: 24 ± 2 mV). Time‐course analysis of the GFP fluorescence intensity revealed a substantial increase in nanoparticle fluorescence over time (Figure S3B). Comparative analysis of @RBCM/cRGDLips and @phLips targeting efficiency in LO2 and HCC‐LM3 cells demonstrated significantly higher uptake by tumor cells, highlighting the robust tumor‐targeting ability of @RBCM/cRGD‐phLips (Figure S3C–F). The nanoparticles underwent significant morphological transformation and size reduction in acidic microenvironments. As the pH decreased from physiological levels (pH 7.4) to tumor‐mimicking acidic conditions (pH 6.2 and 5.0), the nanoparticle structures progressively transitioned from intact spheres to fragmented forms through a gradual disassembly process (Figure 2G,H). Coomassie blue staining confirmed the stability and integrity of the RBCM components in the nanoparticles (Figure 2I,J). Additionally, the phagocytic activity of macrophages towards @RBCM/cRGD‐phLips was significantly inhibited owing to the presence of the glycoprotein CD47 on RBCMs (Figure 2K), which can bind to the signal regulatory protein α on the surface of macrophages, thereby suppressing phagocytosis by macrophages and facilitating immune evasion. Treatment with sgRRM2‐loaded LNPs significantly inhibited RRM2 expression in HCC‐LM3 and SK‐HEP1 cells (Figure 2L,M), and SPIO loading exhibited low cytotoxicity (Figure S3G,H). Using the loaded SPIO for MRI, the results highlighted the superior tumor‐targeting capabilities and diagnostic value of SPIO@RBCM/cRGD‐Lips (Figure S3I,J). Compared to conventional pHLips, RBCM/cRGD‐Lips demonstrated superior tumor targeting and accumulation capabilities, which are essential for the development of cancer‐targeted therapeutic agents (Figure S3K,L). These results confirm the successful preparation of engineered LNPs, which provide effective and stable delivery of *sgRRM2* and SPIO, significantly inhibit macrophage phagocytosis, and further enhance tumor targeting.

### Co‐delivery of SPIO and *sgRRM2* via nanocarriers enhances ferroptosis in HCC

**FIGURE 3:**
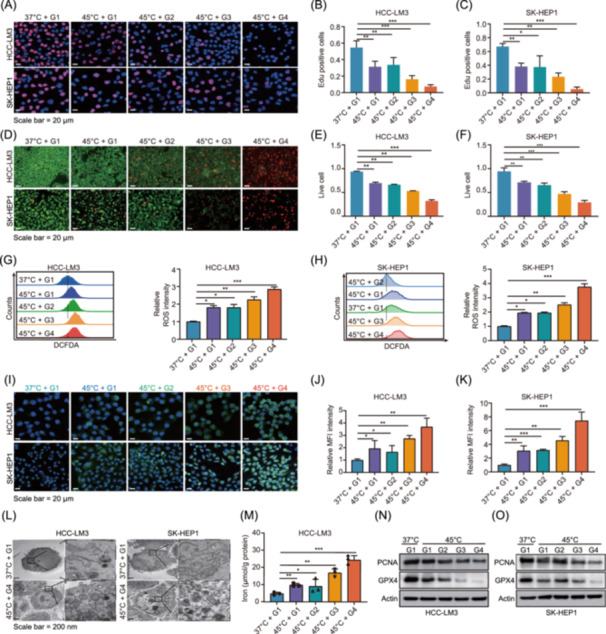
Multifunctional nanoplatform shows that SPIO and *sgRNA* synergistically enhance ferroptosis in HCC. (A) G1, @RBCM/cRGD‐phLips (control); G2, SPIO@RBCM/cRGD‐phLips; G3, sg*RRM2*@RBCM/cRGD‐phLips; G4, sS@RBCM/cRGD‐phLips. Representative IF micrographs of HCC‐LM3 and SK‐HEP1 cells following EdU incorporation assay under five different treatment conditions. (B, C) Quantification of proliferating HCC‐LM3 (B) and SK‐HEP1 (C) cells under five different treatment conditions as determined by the EdU incorporation assay. (D) Viability assessment of HCC‐LM3 and SK‐HEP1 cells under five different treatment conditions using calcein AM (green, viable cells) and Eth‐1 (red, nonviable cells). (E) Quantification of SK‐HEP1 proliferation under various treatment conditions. (F) Percentage of viable SK‐HEP1 cells corresponding to (D). (G, H) Flow cytometric analysis of reactive oxygen species (ROS) levels in HCC‐LM3 (G) and SK‐HEP1 (H) cells after five different treatments, as measured by the intensity of the DCFDA probe. G1 served as the control group. (I) Representative IF micrographs of HCC‐LM3 and SK‐HEP1 cells stained with C11‐BODIPY (a lipid peroxidation indicator) after five treatments. (J, K) Quantitative analysis of the relative mean fluorescence intensity of HCC‐LM3 and SK‐HEP1 cells. (L) Representative TEM images illustrating mitochondrial ultrastructural changes in HCC‐LM3 and SK‐HEP1 cells after combined treatment with SPIO and sg*RRM2*‐loaded nanocarriers. (M) Quantitative analysis of the intracellular ion levels in HCC‐LM3 cells. Western blot analysis of proliferating cell nuclear antigen (PCNA), glutathione peroxidase 4 (GPX4), and actin expression in HCC‐LM3 (N) and SK‐HEP1 (O) cells treated with G1–G5. Actin served as the loading control. Scale bar = 20 μm. *p < 0.05; **p < 0.01; ***p < 0.001.

**FIGURE 4:**
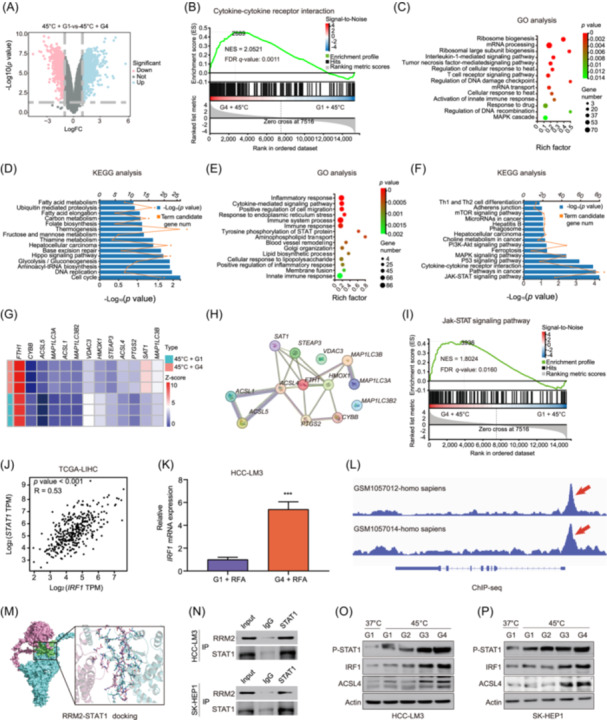
Nanoparticle co‐delivery system upregulates interferon regulatory factor 1 (*IRF1*) and acyl‐CoA synthetase long‐chain family member 4 (*ACSL4*) expression, enhancing ferroptosis and antitumor immune response in HCC. (A) Volcano plot depicting differentially expressed genes (DEGs) after RFA treatment. (B) Gene set enrichment analysis (GSEA) demonstrating RFA‐associated cytokine–cytokine receptor interactions. (C) Gene Ontology (GO) enrichment bubble plots. The *x*‐axis represents the gene ratio, the bubble size indicates the number of genes, and the color intensity correlates inversely with the *p*‐value. (D) Kyoto Encyclopedia of Genes and Genomes (KEGG) pathway analysis. The orange curve represents the number of candidate genes. (E) GO enrichment bubble plot illustrating the significant biological processes. (F) KEGG pathway analysis highlighting the enriched signaling pathways. (G) Stacked‐bar plot depicting the distribution of DEGs after RFA treatment. (H) Protein–protein interaction network visualization of the co‐expressed genes. (I) GSEA results demonstrating the enrichment of the RFA‐associated JAK‐STAT signaling pathway. (J) Scatter plot and Spearman's correlation analysis illustrating the relationship between IRF1 and STAT1 expression. (K) Relative IRF1 mRNA expression in HCC‐LM3 cells. (L) ChIP data from GSM1057012 and GSM1057014 datasets revealing distinct *STAT1* binding peaks. (M) Molecular docking simulations illustrating the interaction between RRM2 and STAT1. (N) Western blot analysis showing RRM2 and STAT1 expression in HCC‐LM3 and SK‐HEP1 cells after Input, IgG, and *STAT1* immunoprecipitation. Western blot analysis showing phosphorylated STAT1 (p‐STAT1), IRF1, ACSL4, and actin expression in HCC‐LM3 (O) and SK‐HEP1 (P) cells under various treatment conditions. ***p < 0.001.

Nanoparticles were categorized into four groups based on their composition: G1, @RBCM/cRGD‐phLips (control); G2, SPIO@RBCM/cRGD‐phLips; G3, sgRRM2@RBCM/cRGD‐phLips; and G4, sS@RBCM/cRGD‐phLips. These groups represent distinct therapeutic strategies for the treatment of HCC. In vitro simulation of RFA‐induced thermal stress was performed by exposing HCC‐LM3 and SK‐HEP1 cells to 45°C. EdU incorporation assays and live–dead staining assays revealed that *RRM2* knockout nanoparticles significantly inhibited tumor cell proliferation and induced cell death, and SPIO and sgRRM2 co‐delivery synergistically enhanced the antitumor effects (Figure 3A–F). Ferroptosis, a regulated form of cell death, is characterized by the accumulation of intracellular iron ions and lipid peroxidation, driven by elevated levels of lipid reactive oxygen species (ROS) and decreased glutathione peroxidase 4 (GPX4) activity [28, 29]. Treatment with dual‐loaded nanoparticles resulted in (1) increased ROS accumulation (Figure 3G,H), which was detected by flow cytometry using the DCFDA fluorescent probe, with the dual‐carrier nanoparticle group (G4) showing ROS fluorescence intensity nearly threefold higher than that in the control group (G1); (2) elevated lipid peroxidation, indicated by C11‐BODIPY fluorescence (Figure 3I–K). C11‐BODIPY is a fluorescent probe that specifically detects lipid peroxidation, transitioning from green to red fluorescence; lipid peroxidation levels in the G4 group were significantly higher than those in the other groups; (3) TEM revealed typical ferroptotic morphological changes in mitochondria of HCC‐LM3 and SK‐HEP1 cells treated with G4, including reduced cristae, increased membrane density, and volume shrinkage (Figure 3L); (4) significant increases in intracellular iron and malondialdehyde (MDA) levels, coupled with decreased GSH content (Figures 3M and S4A–E) Biochemical analysis showed an approximately 2.5‐fold increase in iron ion concentration, a twofold increase in MDA content, and a reduction of GSH levels to approximately 40% that of the control group after G4 treatment, all characteristic biochemical features of ferroptosis; (5) western blot analysis confirmed that G4 treatment significantly downregulated GPX4 protein expression and significantly inhibited proliferating cell nuclear antigen (PCNA) expression, indicating suppressed cell proliferation (Figure 3N,O). These results suggest that SPIO and sgRRM2 co‐delivery via nanoparticles effectively promotes ferroptosis in HCC cells.

Immunogenic cell death (ICD) plays a pivotal role in immune surveillance and cancer immunotherapy [30, 31, 32]. Nanoparticle‐based therapies can markedly augment the efficacy of cancer immunotherapy. Nanoparticles can enhance immunotherapy through multiple mechanisms, including improved antigen delivery, protection of biomolecules from degradation, and controlled release of immunomodulatory agents at tumor sites [33]. *RRM2* knockout combined with RFA significantly promoted the expression of calreticulin (CRT) and high‐mobility group box 1 (HMGB1), two key markers of ICD, suggesting that RRM2 knockout enhanced RFA‐induced ICD, and SPIO and sgRRM2 co‐loading further amplified this effect (Figures S4F–H and S5A–F). These results were validated using western blot analysis, which demonstrated that combined treatment with sS@RBCM/cRGD‐phLips and RFA induced an approximately increase in CRT expression and increase in HMGB1 release compared with RFA monotherapy (Figure S5H,I). Given that DCs are crucial antigen‐presenting cells in the immune system, the percentage of CD80+CD86+ DCs was evaluated in a coculture system containing bone marrow‐derived DCs and Hepa1‐6 cells to assess the effect of ICD on DC maturation (Figure S5G,J). Flow cytometry revealed that in the presence of Hepa1‐6 cells treated with sS@RBCM/cRGD‐phLips combined with RFA, the percentage of CD80+CD86+ DCs reached a maximum of 59.7%, which is more than double that observed with RFA treatment alone. This significant increase in the co‐expression of CD80 and CD86 costimulatory molecules indicates enhanced DC maturation and activation, which is essential for efficient T cell priming and adaptive immune responses (Figure S5K). These results indicate that *RRM2* knockout increases RFA‐induced ICD and activates the DC‐mediated immune response, thereby augmenting the overall antitumor effect.

### Nanoparticle targeting RRM2 upregulates the STAT1‐IRF1‐ACSL4 axis in HCC

Transcriptome sequencing was performed on G4/G1 samples (Figure 4A), followed by gene set enrichment analysis of the upregulated DEGs to elucidate the molecular mechanisms by which *RRM2* regulates HCC progression. The cytokine‐cytokine receptor signaling pathways were significantly enriched (Figure 4B). Gene Ontology and Kyoto Encyclopedia of Genes and Genomes (KEGG) pathway analyses further indicated close correlations between cellular heat‐response regulation and fatty acid metabolism (Figure 4C,D). Analysis of downregulated DEGs revealed significant associations between STAT signaling pathways and ferroptosis‐related processes (Figure 4E,F). sS@RBCM/cRGD‐phLips significantly upregulated acyl‐CoA synthetase long‐chain family (ACSL) expression (Figure 4G,H). Comprehensive gene analysis revealed a strong association between sS@RBCM/cRGD‐phLips treatment and JAK–STAT signaling pathway activation, highlighting a positive correlation between STAT1 and interferon regulatory factor 1 (*IRF1*) (Figure 4I,J). Significant *IRF1* upregulation in HCC‐LM3 cells was detected after treatment with a combination of sS@RBCM/cRGD‐phLips and RFA, and subsequent ChIP‐seq analysis confirmed the presence of binding sites between *STAT1* and *IRF1*, indicating that *IRF1* is a potential downstream target of *STAT1* (Figure 4K,L). Molecular docking simulations also suggested a potential interaction between RRM2 and STAT1 (Figure 4M), which was further validated by immunoprotein assays (Figure 4N). The STAT1/IRF1 axis regulates acyl‐CoA synthetase long‐chain family member 4 (*ACSL4*) [34] and serves as a crucial potential target for inducing ferroptosis [35]. Western blot analysis showed significant phosphorylated STAT1, IRF1, and ACSL4 upregulation in both HCC‐LM3 and SK‐HEP1 cells after treatment with a combination of sS@RBCM/cRGD‐phLips and RFA (Figure 4O,P). These findings indicate that the nanoparticle co‐delivery system enhances ferroptosis via *RRM2* knockout by activating the STAT1–IRF1–ACSL4 axis, thereby amplifying the antitumor effects in HCC.

### Nanoparticle co‐delivery system potentiates the antitumor efficacy of RFA in HCC with minimal toxicity

**FIGURE 5:**
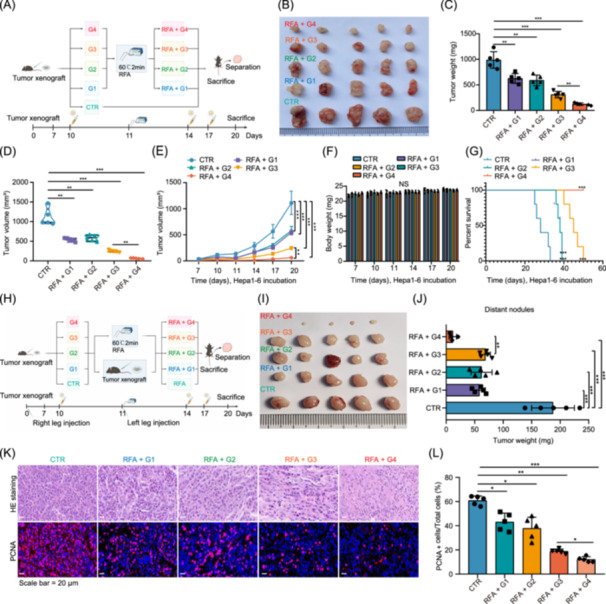
Dual‐loaded nanoparticle co‐delivery system potentiates the antitumor efficacy of RFA in HCC while maintaining a favorable safety profile. (A) Flowchart of the animal model illustrating the treatment timeline and nanoparticle co‐delivery system administration following RFA (*n* = 5). (B) Comparative analysis of tumor sizes under various post‐RFA treatment conditions showing a significant tumor size reduction in the RFA + G4 nanoparticle group. (C) Histogram comparing tumor weights across treatment groups, demonstrating the lowest tumor weight in the RFA + G4 nanoparticle group. (D) Violin plot showing the distribution of tumor volumes in each treatment group, with a more concentrated and smaller volume in the RFA + G4 nanoparticle group. (E) Line graph depicting tumor growth kinetics showing slower tumor growth in the RFA + G4 nanoparticle group than in the other groups. (F) Histogram showing changes in body weight, indicating minimal weight loss across all groups, reflecting a favorable safety profile. (G) Line graph of survival rates demonstrating prolonged survival in the RFA + G4 nanoparticle group compared to the other treatments. (H, I) Tumor size images under different treatments showing visibly smaller tumors in the RFA + nanoparticle group (*n* = 5). (J) Histogram summarizing tumor weight statistics confirming a significant tumor weight reduction in the RFA + G4 nanoparticle group. (K) Representative hematoxylin and eosin (H&E) staining images of HCC tumor tissues showing extensive structural destruction in the RFA + G4 nanoparticle group, along with IF staining for proliferating cell nuclear antigen (PCNA)‐positive cells (a proliferation marker), where PCNA is shown in red and cell nuclei are shown in blue (DAPI). (L) Quantitative analysis of PCNA‐positive cells from IF images revealing a significant reduction in proliferation in the RFA + G4 nanoparticle group. Scale bar = 20 μm. *p < 0.05; **p < 0.01; ***p < 0.001.

In vitro experiments demonstrated that co‐loading SPIO nanoparticles with *RRM2* knockout significantly enhanced antitumor effects. In vivo experiments were then performed, designating G1 as the control group and G2–G4 as the treatment groups. After establishing the tumor model, the first nanoparticle treatment was administered on Day 10 (1 day before RFA), followed by RFA treatment on Day 11, with subsequent nanoparticle treatments on Days 14 and 17 (3 and 6 days after RFA). Compared with the control group, the treatment groups exhibited significant knockout of tumor size, weight, volume, and growth rate. Compared with the antitumor effects of the nanoparticles in the control group and SPIO nanoparticles, the *sgRRM2* nanoparticles showed significantly higher antitumor efficacy than the SPIO nanoparticles. Notably, the dual‐loaded nanoparticles significantly amplified the antitumor effect (Figure 5A–E). Furthermore, no significant changes in body weight were observed in any of the groups (Figure 5F). Histological analysis revealed no significant structural alterations in the tissues across all the groups (Figure S6A). Liver function indicators (alanine aminotransferase [ALT], aspartate aminotransferase [AST], and albumin [ALB]), myocardial enzyme markers (creatine kinase [CK]), and renal function parameters (creatinine [CR] and blood urea nitrogen [BUN]) remained stable (Figure S6B–G), demonstrating the excellent biocompatibility of the nanomaterials. Multiplex cytokine arrays revealed increased C–C motif chemokine ligand 24 (CCL24) and CCL2 secretion (Figure S6H,I), indicating that RFA treatment combined with *RRM2* knockout induces robust antitumor immune activation. Additionally, dual‐loaded nanoparticles significantly prolonged mouse survival (Figure 5G). A metastatic Hepa1‐6 tumor ablation model was developed to further elucidate the antitumor immune effects of the dual‐loaded nanoparticles in HCC (Figure 5H). The tumor size, weight, and volume were significantly reduced, with the dual‐loaded nanoparticles exhibiting the most pronounced antitumor effects (Figure 5I–L). These results highlight the prominent advantage of the nanoparticle co‐delivery system in enhancing the antitumor efficacy of RFA targeting HCC.

### Nanoparticle co‐delivery system enhances antitumor immunity and modulates the TME

**FIGURE 6:**
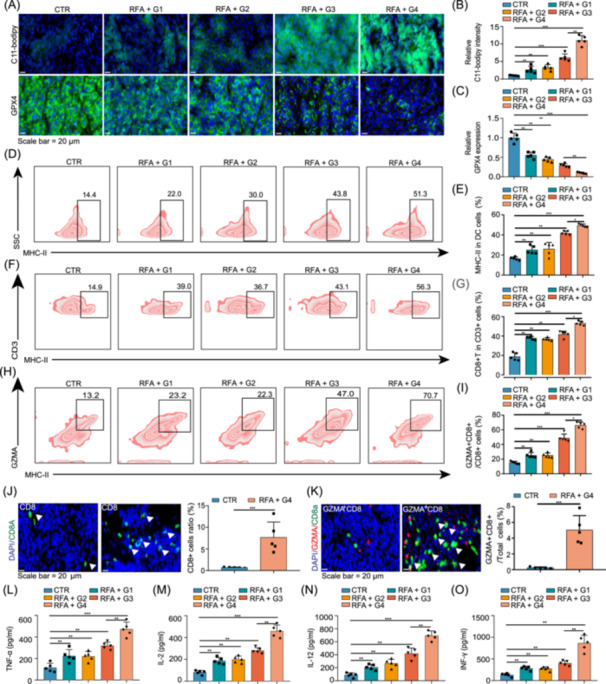
Co‐delivery system of dual‐loaded nanoparticles activates antitumor immunity and affects the immune microenvironment. (A) Representative C11‐BODIPY staining showing lipid peroxidation levels in HCC tumor tissues, indicating increased oxidative stress in the co‐delivery system group. (B, C) Relative levels of malondialdehyde (MDA), a marker of lipid peroxidation, in HCC tumor tissues, demonstrating significantly elevated MDA levels in tumors treated with the nanoparticle co‐delivery system, indicating enhanced ferroptosis. (D) Representative false‐color flow cytometry plots of MHC‐II expression gated to live cells in HCC tumor tissues showing a progressive increase in the proportion of MHC‐II‐positive cell populations across different treatment groups (control [CTR], RFA + G1–G4), with percentages of 14.4%, 22.0%, 30.0%, 43.8%, and 51.3%, respectively, suggesting enhanced activation of antigen‐presenting cells. The red regions and contour lines in the figure represent the distribution of cell numbers, with darker red or denser contour lines indicating a higher number of cells. (E) Statistical histogram quantifying the percentage of MHC‐II+ DCs. Higher percentages were observed in the co‐delivery system group, suggesting enhanced DC activation. (F) False‐color flow cytometry plots for CD8 and CD3 expression in HCC tumor tissues gated to live cells, showing increased CD8+ T cell infiltration in the co‐delivery system group with a gating strategy based on CD8 and CD3 co‐expression, showing significantly increased proportions of CD3+CD8+ cells in different RFA combination treatment groups (from 14.9% in the control group to 56.3% in the RFA + G4 group). These results indicate the effective activation of cytotoxic T cells. (G) Statistical histogram of CD8+ T cells as a proportion of CD3+ cells, confirming enhanced recruitment of cytotoxic T cells. (H) Flow cytometry plots showing CD8 and granzyme A (GZMA) expression in live tumor cells, with higher levels of GZMA‐positive CD8+ T cells in the co‐delivery system group. (I) Statistical analysis of GZMA+CD8+ T cells as a percentage of total CD8+ cells showing enhanced cytotoxic activity in the co‐delivery system group. (J) Demonstrates the quantitative analysis of GZMA+CD8+ cell percentages across different treatment groups (from CTR to RFA + G4), with data showing that as treatment intensity increases, the proportion of CD8+ cells expressing granzyme A significantly rises from approximately 20% in the control group to about 70% in the RFA + G4 group. (K) Provides a visual demonstration of the distribution differences of CD8+ T cells between the control group and the RFA + G4 group through immunofluorescence staining, with the fluorescence images on the left showing significantly increased CD8‐positive cells (marked by white arrows) in the RFA + G4 group, while the quantitative analysis on the right further confirms that the percentage of CD8‐positive cells in the treatment group is significantly higher than in the control group. (L–O) Levels of key cytokines in tumor tissues, including tumor necrosis factor‐alpha (TNF‐α) (L), interleukin‐2 (IL‐2) (M), interleukin‐12 (IL‐12) (N), and interferon‐gamma (IFN‐γ) (O), showing significant upregulation in the co‐delivery system group and reflecting enhanced antitumor immune responses. Scale bar = 20 μm. *p < 0.05; **p < 0.01; ***p < 0.001.

The effect of the nanoparticle co‐delivery system on the regulation of the TME was further explored. sS@RBCM/cRGD‐phLips significantly amplified the RFA‐induced lipid peroxidation upregulation and GPX4 inhibition (Figure 6A–C), confirming that the nanoparticle co‐delivery system potentiated RFA‐induced ferroptosis and ICD. Flow cytometric analysis revealed a progressive increase in immune activation markers across the treatment groups. Specifically, MHC‐II+ DC increased from 14.4% in the control group to 51.3% in the RFA + G4 group (Figure 6D,E), with significant differences between consecutive treatment groups. Similarly, CD3+ CD8+ T cell populations expanded substantially from 14.9% in the control to 56.3% in the RFA + G4 group (Figure 6F,G), indicating robust T cell recruitment and activation. The most dramatic enhancement was observed in granzyme A (GZMA)+CD8+ cytotoxic T cells, which increased from 13.2% in the control group to 70.7% in the RFA + G4 group (Figure 6H,I), representing a 5.4‐fold increase in the number of cells with direct tumor‐killing capacity. Immunofluorescence assays further confirmed that treatment with the nanocarrier system significantly increased both GZMA+CD8+ double‐positive cells and CD8+ T cells (Figure 6J,K). The immunofluorescence images in panels J and K provide visual confirmation of these findings, with white arrowheads highlighting the increased presence of CD8+ T and GZMA+CD8+ double‐positive cells in tumor tissues from the RFA + G4 group compared to the control group. The adjacent bar graphs quantify this increase, showing approximately 8–10‐fold higher numbers of these effector cells under the RFA + G4 treatment condition. Concurrently, RFA‐induced upregulated levels of antitumor cytokines, including interleukin‐2, interleukin‐12, and interferon‐gamma, were significantly elevated after treatment with the nanocarrier system (Figure 6L–O). These findings demonstrate that the nanoparticle co‐delivery system effectively activates and enhances RFA‐induced in vivo immune responses and increases antitumor effects.

Ferrostatin‐1 (Fer‐1), a potent ferroptosis inhibitor that specifically prevents lipid peroxidation and subsequent ferroptotic cell death by acting as a radical‐trapping antioxidant, was used to elucidate the role of the nanocarrier co‐delivery system in inhibiting tumor progression. Fer‐1 treatment significantly reversed the inhibitory effects of the nanocarrier co‐delivery system on tumor size, weight, and growth rate compared to the control group (Figure S7A–E). No significant biotoxicity was observed during the experiment (Figure S7G). Although the nanoparticle co‐delivery system significantly inhibited tumor proliferation and progression, this effect was attenuated by Fer‐1 treatment (Figure S7F–H). A mouse model of orthotopic liver cancer transplantation was constructed, in which Fer‐1 treatment significantly impeded the antitumor effects of the nanocarrier co‐delivery system (Figure S7I–L). These findings provide strong evidence that the nanoparticle co‐delivery system exerts antitumor effects via ferroptosis induction.

### Targeting *RRM2* nanoparticle co‐delivery system combined with RFA treatment augments anti‐PDL1 efficacy in HCC

**FIGURE 7:**
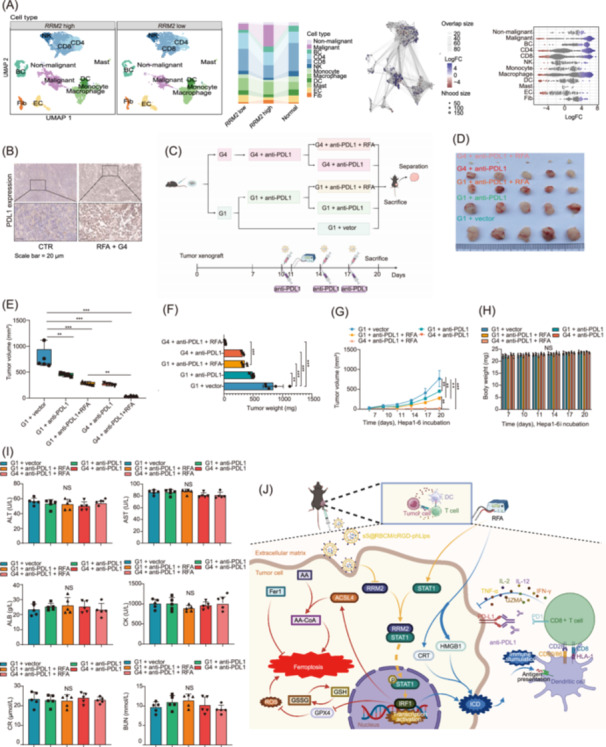
Dual‐loaded nanoparticle co‐delivery system enhances the immune effect of HCC. (A) Comparison of cellular composition and abundance in HCC tumor tissues between the *RRM2* high and *RRM2* low groups, showing increased proportions of CD8+ T cells and DCs, along with decreased malignant cell abundance in the *RRM2* low group, highlighting the enhanced immune microenvironment with *RRM2* downregulation. (B) Programmed cell death protein 1 (PDL1) expression analysis in HCC tumor tissues with or without RFA treatment, indicating reduced PD‐L1 expression in tumors treated with the dual‐loaded nanoparticle co‐delivery system, suggesting immune checkpoint modulation. (C) Treatment scheme for Hepa1‐6 tumor‐bearing mouse models, illustrating the experimental timeline and interventions. (D) Tumor size comparisons under different treatment conditions, with significant tumor size reduction observed in the RFA + anti‐PDL1 + nanoparticle group. (E) Histogram comparing tumor weights across groups, demonstrating the lowest tumor weight in the RFA + anti‐PDL1 + nanoparticle group. (F) Statistical box plot of tumor volume in each group, showing consistent tumor volume reduction in the dual‐loaded nanoparticle‐treated group. (G) Line graph depicting tumor growth kinetics, with markedly slower tumor growth in the RFA + anti‐PDL1 + nanoparticle group compared to other treatments. (H) Statistical histogram of mouse body weight changes, indicating stable body weight and minimal systemic toxicity across all treatment groups. (I) Blood biochemical analysis showing alanine aminotransferase (ALT), glutamate aminotransferase (AST), albumin (ALB), creatine kinase (CK), creatinine (CR), and urea nitrogen (BUN) levels, with no significant toxicity observed in the dual‐loaded nanoparticle‐treated group. (J) Mechanism diagram summarizing the antitumor immune‐enhancing effects of the dual‐loaded nanoparticle co‐delivery system, including *RRM2* knockout, ferroptosis induction, and immune microenvironment modulation through increased CD8+ T cell infiltration and reduced PDL1 expression. Scale bar = 20 μm.

*RRM2* downregulation enhances the efficacy of immunotherapy. To further elucidate the effect of *RRM2* downregulation on immune efficacy, scRNA‐seq data were used to explore the influence of *RRM2* on the TME in HCC. The TME composition was determined based on *RRM2* expression levels in the epithelial cells of the tumor tissues, which were categorized into *RRM2* high and *RRM2* low groups. Notable, compared to the *RRM2* high group, the *RRM2* low group exhibited significantly higher proportions and cellular abundance of CD8+ T and DCs, along with a marked reduction in malignant cells (Figure 7A). This observation revealed a complex interplay between *RRM2* expression and the immune landscape within the HCC microenvironment. The increase in CD8+ T cells and DCs in the *RRM2* low group suggests enhanced immune infiltration and activation, potentially contributing to a more robust antitumor immune response. Concurrently, the reduction in the number of malignant cells in the *RRM2* low group aligns with previous findings regarding the potential therapeutic benefits of *RRM2* downregulation.

Immunohistochemistry revealed elevated programmed cell death protein 1 (PDL1) expression, suggesting that PDL1 inhibition increased the antitumor effects of the nanoparticle co‐delivery system (Figure 7B). The application of anti‐PDL1 antibodies to the tumor model demonstrated that combination therapy with anti‐PDL1 and the nanoparticle co‐delivery system suppressed HCC progression (Figure 7C–G). Furthermore, this combination therapy exhibited favorable biocompatibility (Figure 7H). No significant alterations were observed in murine liver function markers (ALT, AST, and ALB), cardiac enzyme levels (CK), or renal function parameters (CR and BUN) (Figure 7I). This study revealed that *RRM2* is overexpressed in tumors and is negatively correlated with patient prognosis. *RRM2* knockout potentiates the antitumor effects of RFA by amplifying both ICD and ferroptosis (Figure 7J). The optimized nanoparticle system further augmented the immune response and enhanced antitumor efficacy.

## DISCUSSION

HCC is the most prevalent malignancy and is characterized by its subtle onset and late‐stage diagnosis in most cases [36, 37]. Despite an array of current therapeutic modalities for HCC, innovative treatment strategies to enhance patient survival are lacking. *RRM2* is a promising therapeutic target for HCC, and its knockout offers an effective approach to combat this disease [10, 13]. Accumulating evidence suggests that downregulating *RRM2* expression has significant therapeutic efficacy in various cancer models [38, 39]. This study revealed a significant correlation between high *RRM2* expression and poor prognosis in patients with HCC and that *RRM2* knockout significantly impaired HCC proliferation. RBCM‐modified nanoparticles (@RBCM/c‐RGD‐phLips) encapsulating *sgRRM2* were engineered to augment in vivo antitumor efficacy by inhibiting *RRM2* expression using *sgRRM2* and enhanced tumor cell‐targeting capabilities were observed. Furthermore, RFA was incorporated into the experimental paradigm, leveraging its safety profile, minimal invasiveness, and potential to expedite patient recovery. Integrating nanomaterials with RFA and immunotherapy is a promising trend in cancer treatment [40], as rationally designed nanomaterials, in conjunction with immunotherapeutic approaches, have demonstrated enhanced tumor treatment efficacy.

RFA is currently the preferred ablation technique for the treatment of liver cancer, particularly for small primary or metastatic hepatic lesions. RFA offers numerous advantages, including operability, safety, and high efficacy, while also modulating the tumor immune microenvironment, thereby enhancing overall cancer treatment outcomes [41]. In this study, *RRM2* expression was significantly upregulated in Hepa1‐6 nodule tissues after RFA treatment, and *RRM2* knockout not only significantly suppressed tumor cell proliferation but also promoted tumor cell ferroptosis, offering a dual mechanism of action against HCC. To gain further insight into the underlying mechanism, the STAT1‐IRF1‐ACSL4 axis was confirmed as a crucial pathway for *RRM2* to activate ferroptosis. *RRM2*‐mediated ferroptosis involves multiple crucial upstream and downstream signaling pathways. Upstream, *RRM2* inhibits ferroptosis by regulating GSH levels, and its knockout leads to decreased GSH levels, weakening cellular antioxidant capacity and promoting ferroptosis induction. Additionally, *RRM2* knockout activated the STAT1‐IRF1 axis, a signaling pathway that plays a pivotal role in ferroptosis, further enhancing lipid peroxidation and ferroptosis sensitivity. Downstream, *RRM2* knockout drives ferroptosis by upregulating *ACSL4* expression, thereby increasing the production of lipid peroxidation substrates. Iron ion accumulation and ROS generation exacerbate lipid peroxidation, ultimately leading to cell death. These mechanisms not only directly enhance the therapeutic effect of RFA but also improve antitumor immune responses by remodeling the TME and activating ICD, providing novel strategies for HCC treatment. This study provides compelling evidence that the combination of *RRM2* targeting and RFA represents a promising therapeutic strategy, potentially leading to substantially improved treatment outcomes in HCC. Integrating *RRM2*‐targeted nanoparticles with RFA exhibits substantial clinical potential, particularly in addressing the limitations of RFA monotherapy. Although RFA demonstrates efficacy in local tumor ablation, it frequently fails to eliminate residual tumor cells or prevent recurrence owing to incomplete ablation and the immunosuppressive TME. Engineered nanoparticles (sS@RBCM/cRGD‐phLips) overcome these challenges through the tumor‐specific delivery of *RRM2* sgRNA, enhancing RFA‐induced ferroptosis and ICD. This dual approach improves local tumor control and activates systemic antitumor immunity, providing a comprehensive therapeutic strategy for patients with HCC. The integration of engineered nanoparticles with RFA therapy presents a novel direction for the precise treatment of HCC. Through enhanced thermal effects, optimized antitumor immune responses, and combination with immunotherapy, this strategy shows promise for improving survival outcomes in patients with cancer. Future research should explore nanoparticle optimization, targeted delivery mechanisms, and clinical safety profiles to facilitate the translation of this combinatorial approach from laboratory to clinical practice, ultimately providing more efficient and safer therapeutic solutions for cancer treatment.

The integration of cancer nanomedicine with immunotherapy has demonstrated significant potential for the advancement of cancer treatment strategies. PDL1 is a pivotal immune checkpoint molecule that, when inhibited, enhances the recognition and subsequent elimination of tumor cells by the host immune system. PDL1 expression in tumor cells plays a crucial role in the evasion of immune surveillance, contributing to cancer progression and immunotherapeutic resistance. Strategies targeting PDL1, including those promoting its degradation, significantly enhance the efficacy of cancer immunotherapy [42, 43, 44]. Although monotherapy targeting PDL1 has shown promise, combination therapies incorporating PDL1 inhibition have emerged as a focal point in contemporary cancer treatment research, offering the potential for improved therapeutic outcomes. RFA not only attenuates tumor progression but also remodels the TME, thus enhancing DC, CD4+, and CD8+ T lymphocyte infiltration and upregulating PDL1 expression [45]. The induction of ferroptosis and concomitant activation of the TME during RFA treatment synergistically augments tumor growth inhibition [46]. The results of this study revealed that dual‐loaded nanoparticles potentiated ferroptosis and modulated the TME, promoting MHC II+ DC and CD8+ T cell recruitment and infiltration, especially GZMA+CD8+ T cells, thereby exerting a robust antitumor effect. *RRM2* knockout significantly increased the immunomodulatory effects of RFA on HCC, offering an efficacious antitumor treatment strategy by amplifying and sustaining the RFA‐induced antitumor immune response. This immune stimulation augments tumor antigen presentation and activates DCs and T lymphocytes, thereby potentiating the post‐RFA immune response and mitigating the escape of residual tumor cells. Mechanistically, the upregulation of key ferroptosis markers, including increased lipid peroxidation and decreased GPX4 expression, correlated with enhanced ICD markers, such as HMGB1 and CRT. Moreover, modified dual‐loaded nanoparticles significantly enhanced RFA‐induced PDL1 upregulation, and the combinatorial approach of targeting ferroptosis and modulating the TME in conjunction with PDL1 inhibition significantly enhanced RFA efficacy and suppressed HCC recurrence and progression.

Despite the compelling evidence provided by this study on *RRM2*‐targeted therapy combined with RFA for HCC treatment, several limitations need to be addressed in future research. While we achieved significant therapeutic effects using the *CRISPR/Cas9* system targeting the *RRM2*, potential off‐target modifications from *CRISPR/Cas9* could lead to unintended alterations in gene function, affecting the safety and specificity of the treatment. For future clinical translation research, it is crucial to adopt more comprehensive whole‐genome analysis methods to systematically evaluate the specificity of *sgRRM2*. Additionally, this study primarily focused on short‐term therapeutic effects and toxicity assessment of the nanocarrier system, with insufficient investigation into its long‐term biocompatibility, immunogenicity, and the effects of prolonged *RRM2* knockout. Particularly, RBCM‐modified nanoparticles might induce immune responses in long‐term applications, necessitating extended safety studies. Despite these limitations, the *RRM2*‐targeted nanocarrier system demonstrated in this study still provides a valuable strategy for innovative HCC treatment, especially in enhancing RFA efficacy and modulating the tumor immune microenvironment. Nevertheless, this study systematically evaluated the therapeutic efficacy of a rationally designed dual‐loaded nanoparticle platform in combination with RFA, using multiple in vivo experimental models. Through a rigorous preclinical assessment, remarkable synergistic antitumor effects were observed when this combinatorial approach was integrated with PDL1 checkpoint blockade. Notably, experimental data demonstrated significantly enhanced tumor growth inhibition and improved survival outcomes compared with individual treatment modalities. The strategic integration of PDL1 checkpoint inhibition, RFA‐induced ICD, and the sophisticated nanoparticle‐based delivery platform resulted in a synergistic therapeutic paradigm. This multifaceted approach effectively addresses multiple aspects of tumor biology, including direct tumor cell killing, immune system activation, and TME modulation. The engineered nanoplatform demonstrated superior tumor‐targeting capabilities and controlled release properties, enabling optimal therapeutic efficacy while minimizing systemic adverse events. This novel combinatorial therapeutic strategy not only presents promising avenues for future oncological interventions but also exhibits substantial potential for clinical translation and application.

## CONCLUSION

This study elucidated the tumor‐suppressive effects of *RRM2* knockout and introduced an innovative nanoparticle formulation, sS@RBCM/cRGD‐phLips. Using a nanoparticle‐based delivery platform, the synergistic combination of *sgRRM2* and SPIO significantly increased the sensitivity of HCC to RFA. Future studies should focus on further refining the nanoparticle design and integrating it with complementary therapeutic modalities to achieve a more comprehensive and robust antitumor response. The findings of this study demonstrated that *sgRRM2*‐loaded nanoparticles exhibited enhanced tumor‐targeting specificity and superior antitumor functionality compared with their unloaded counterparts, with significantly improved therapeutic efficacy when combined with RFA. This innovative strategy, which integrates nanotechnology with gene editing techniques, not only presents broad applicability in HCC management but also opens new avenues for treating various solid tumors.

## METHODS

### Chemicals and reagents

The following chemicals and reagents were used in this study: trichloromethane, methanol, phosphate‐buffered saline (PBS), human LM3 cells, SK‐HEP1 cells, mouse Hepa1‐6 cells, dimethyl sulfoxide (DMSO), 10% fetal bovine serum (FBS), trypsin, 0.3% Triton‐X‐100 in PBS solution, 4% paraformaldehyde solution, Click Additive Solution, 10 µg/mL 4′,6‐diamidino‐2‐phenylindole (DAPI) solution, Calcein‐AM and Ethidium Homodimer‐1 (Eth‐1) mixed solution, paraffin, anti‐quenching mounting agent, TRIzol reagent, microcentrifuge tubes, chloroform, isopropanol, ethanol, reverse transcription kit, 1% RIPA lysis buffer, materials for sodium dodecyl sulfate‐polyacrylamide gel electrophoresis (SDS‐PAGE), Tris‐buffered saline with Tween 20 (TBST) containing 5% skim milk, enhanced chemiluminescence (ECL) reagent, chemiluminescence reagent kit, plate reader, culture medium supplemented with 10 μmol/L Erastin, Cell Counting Kit‐8 (CCK‐8) solution, MDA content detection kit, GSH content detection kit, iron ion content detection kit, 1% agarose solution, liposome extruder (Avanti), 200 nm pore polycarbonate membrane, transmission electron microscope, and fluorescence microscope. 1,2‐Distearoyl‐sn‐glycero‐3‐phosphoethanolamine (DSPE) (R‐PL2056‐1g), Ruixibio, SPIO (R‐C1002, Ruixibio), 1,2‐Dioleoyl‐3‐trimethylammonium‐propane (DOTAP) (LP‐RA‐31T, Ruixibio), 1,2‐Distearoyl‐sn‐glycero‐3‐phosphoethanolamine‐polyethylene glycol‐cyclic RGD peptide (DSPE‐PEG‐cRGD) (R‐8985‐50mg, Ruixibio), Cyanine 5.5 fluorescent dye (CY5.5) (R‐CY3012, Ruixibio).

### Animal models

Six‐to‐eight‐week‐old C57BL/6 or nude male mice were maintained under specific pathogen‐free conditions at 24 ± 2°C with 40%–70% relative humidity and a 12‐h light/dark cycle. Two subcutaneous tumor models were established, with each group containing a minimum of five mice. The first model was developed by subcutaneously injecting 1 × 10^6^ Hepa1‐6 cells in 100 µL into the left lower extremity of 8‐week‐old male C57BL/6 mice. The second model was created by subcutaneously injecting 1 × 10^6^ HCC‐LM3 or Hepa1‐6 cells in 100 µL into the left lower extremity of 8‐week‐old male nude mice. When tumors reached approximately 100 mm^3^, an 18G bipolar ablation needle was inserted into the tumor tissue, connected to a radiofrequency transmitter, set to 60°C, and RFA was performed for 2 min. We isolated bone marrow cells from femurs and tibias of C57BL/6 mice and seeded them at a density of 2 × 10^6^ cells/dish in culture medium containing 10% fetal bovine serum, supplemented with 20 ng/mL recombinant murine GM‐CSF to induce BMDC differentiation. On Days 3 and 5 of culture, 50% of the medium was replaced with fresh medium containing GM‐CSF. Cells were cultured for a total of 6 days.

### Nanocarrier preparation and characterization

Predetermined amounts of SPIO and *sgRRM2* were dissolved in a trichloromethane and methanol mixture. The solution was placed in a round‐bottomed flask and evaporated for 20 min to form a thin film. The film was then dissolved in PBS and sonicated on ice for 10 min. The resulting solution was extruded through a polycarbonate membrane with 200 nm pores using a liposome extruder (Avanti). The product was centrifuged at 10,000 × *g* for 10 min to yield co‐loaded SPIO/*sgRRM2*‐phLips pH‐sensitive liposomes (sS@‐Lvs).

Red blood cells were washed with cold PBS and centrifuged to remove any impurities. The cells were then transferred to a hypotonic buffer for lysis and hemolysis. Hemoglobin and other cellular components were removed to obtain empty red blood cell ghosts. The ghosts were ultrasonically fragmented and dispersed in a PBS suspension containing the nanospheres. The mixture was extruded through a polycarbonate membrane with a pore size of 200 nm using a liposome extruder to form vesicles camouflaged with RBCMs. The extrusion process was repeated to ensure the formation of uniform RBCM‐camouflaged nanoparticles.

Predetermined amounts of cRGD and RBCM were freeze‐thawed three times in liquid nitrogen at room temperature. The mixture was centrifuged at 700 × *g* at 4°C for 10 min, and the supernatant was further centrifuged at 12,000 × *g* at 4°C for 10 min to obtain RBCM/cRGD. Predetermined amounts of SPIO/*sgRRM2*@Lvs and RBCM/cRGD were suspended in PBS and sonicated in an ice bath for 10 min to prepare the SPIO and sgRRM2 co‐loaded RBCM/cRGD‐sensitive liposome fusion vesicles (sS@RBCM/cRGDLvs). The mixture was extruded multiple times using a liposome extruder and centrifuged at 10,000 × *g* for 10 min to obtain sS@RBCM/cRGDLvs.

The zeta potential of the nanoparticles was measured using a zeta potential analyzer, and their morphologies were observed using a transmission electron microscope. All experiments were performed in triplicates. The drug‐loaded nanovesicles were disrupted using DMSO, and their contents were analyzed using UV spectrophotometry. The encapsulation efficiency (EE%) and drug loading (DL%) were calculated. The absorbances of SPIO and sgRRM2 were measured at room temperature using a UV‐visible spectrophotometer. The EE% and DL% were calculated using the following formulas: EE (%) = (*W1*/*W2*) × 100%, DL (%) = (*Wd*/*Wn*) × 100%, where *W1* is the mass of the drug loaded into the nanovesicles, *W2* is the total mass of the drug used, *Wd* is the amount of drug encapsulated in the nanovesicles, and *Wn* is the total mass of the nanovesicles. All experiments were performed in triplicates.

### Cell culturing and passaging

The HCC cell lines LM3 and SK‐HEP1 and the mouse HCC cell line Hepa1‐6 were thawed from liquid nitrogen storage by gentle agitation in a 37°C water bath. The thawed cell suspension was centrifuged at 800 rpm (approximately 200 × *g*) for 10 min to remove the cryopreservation medium. The cell pellet was resuspended in 1 mL of complete culture medium supplemented with 10% FBS. The cell suspension was gently transferred to a culture dish and incubated in a humidified atmosphere of 5% CO_2_ at 37°C. Human HCC cell lines (HCC‐LM3 and SK‐HEP1) and mouse HCC cells (Hepa1‐6) were cultured in complete Dulbecco's Modified Eagle Medium (DMEM). The cells were subcultured to 80%–90% confluence. The culture medium was aspirated, the cells were washed with PBS, and an appropriate volume of 0.25% trypsin‐EDTA solution was added. Cell detachment was monitored using an inverted microscope until the cells became round. Trypsinization was halted by adding a complete culture medium containing 10% FBS. The cell suspension was gently pipetted to obtain a single‐cell suspension. An appropriate number of cells was seeded into new culture dishes containing fresh medium. Cells were maintained in a humidified 5% CO_2_ incubator at 37°C. For cryopreservation, cells at 80% confluence were harvested, resuspended in a freezing medium (90% FBS and 10% DMSO), aliquoted into cryovials, and gradually cooled to −80°C using a freezing container before being transferred to liquid nitrogen for long‐term storage.

### Cell EdU experiment

HCC‐LM3 and SK‐HEP1 cells were seeded in six‐well plates at a density of 2 × 10^5^ cells per well and cultured in complete DMEM supplemented with 10% FBS and 1% penicillin–streptomycin at 37°C in a humidified atmosphere containing 5% CO_2_ until reaching 70%–80% confluence. After washing twice with sterile PBS, the cells were incubated with 10 μM EdU solution (diluted in complete DMEM) for 3 h under standard culture conditions.

The cells were then fixed with a 4% paraformaldehyde solution for 20 min at room temperature and washed three times with PBS. Cell membrane permeabilization was achieved using 0.3% Triton X‐100 in PBS for 15 min at room temperature, followed by three PBS washes and fresh preparation, according to the manufacturer's instructions (Click‐iT^TM^ EdU Cell Proliferation Kit, Thermo Fisher Scientific). Briefly, the reaction mixture contained 430 μL of 1X Click‐iT reaction buffer, 20 μL of CuSO_4_, 1.2 μL of Alexa Fluor Azide, and 50 μL of reaction buffer additive. A 500 μL aliquot of this reaction mixture was added to each well, and the plates were incubated in the dark for 30 min at room temperature with gentle shaking. After three PBS washes, nuclear counterstaining was performed using a DAPI solution (10 μg/mL) for 10 min at room temperature in the dark. Following the final PBS wash, the cells were maintained in 2 mL of fresh PBS for imaging. EdU‐positive cells (green fluorescence) and DAPI‐stained nuclei (blue fluorescence) were visualized and photographed using a fluorescence microscope with appropriate filter sets (wavelengths: EdU = 488 nm, DAPI = 405 nm). At least five random fields per well were used for quantitative analysis.

### Live‐dead cell staining

HCC‐LM3 and SK‐HEP1 cells were seeded in 35 mm glass‐bottom dishes at a density of 2 × 10^5^ cells per dish and cultured in complete DMEM supplemented with 10% FBS and 1% penicillin–streptomycin until reaching 70%–80% confluence. The staining solution was freshly prepared by adding 20 μL Calcein‐AM (2 mM stock solution, Invitrogen) and 5 μL Eth‐1 (1 mg/mL stock solution, Sigma‐Aldrich) to 10 mL serum‐free DMEM, resulting in final concentrations of 4 μM Calcein‐AM and 0.5 μg/mL Eth‐1. After washing twice with sterile PBS, 1 mL of the prepared calcein‐AM/PI staining solution was added to each dish. The cells were incubated at 37°C in a humidified atmosphere containing 5% CO_2_ for 15 min in the dark. After incubation, the cells were gently washed 3–5 times with PBS to remove excess dye. Finally, 1 mL of fresh, complete DMEM was added to each dish. Cell viability was immediately assessed using a fluorescence microscope with appropriate filter sets.

### Subcutaneous tumor and distant metastasis models

Mouse HCC Hepa1‐6 cells were cultured in DMEM and washed with PBS. The cells were digested with trypsin containing EDTA, neutralized with DMEM, transferred to a 15 mL centrifuge tube, and centrifuged at 800 rpm for 10 min. PBS was added and gently pipetted to resuspend the cells, and the cells were counted. The concentration of mouse‐derived HCC Hepa1‐6 cells was adjusted to 1 × 10^6^ cells/mL. The experimental mice were anesthetized with an anesthetic machine, and 200 μL of cell suspension was subcutaneously injected. When the subcutaneous tumors in the mice grew to a diameter of approximately 3 mm, the body weight of the mice was recorded every 3 days, and the length (*L*) and width (*W*) of the tumors were measured. Volume of the subcutaneously transplanted tumors in mice (*V*) = (*L* × *W* ^2^)/2. Once the tumors reached a certain volume, they were removed and photographed, and some tumor tissues were fixed in 4% paraformaldehyde while the remaining tissues were stored at −80°C for future experiments.

### Tissue hematoxylin and eosin staining, immunohistochemistry staining, and immunofluorescence

Mouse tissues were fixed with 4% paraformaldehyde, dehydrated, cleared in xylene, embedded in melted paraffin, sectioned, and stained with hematoxylin and eosin after removing the paraffin. The paraffin sections of mouse Hepa1‐6 tumor tissues were baked in a 65°C constant temperature oven for 1 h, deparaffinized twice with xylene, dehydrated with alcohol, washed with PBS, and subjected to antigen retrieval. The sections were then washed again with PBS, blocked with 5% goat serum solution for 1 h, and incubated with the primary antibody solution overnight at 4°C. The cells were washed with PBS, incubated with the secondary antibody solution at 37°C for 1 h, washed with PBS, and incubated with DAB chromogenic solution for approximately 5 min. After that, they were washed with PBS, stained with hematoxylin for 5 min, washed again, dehydrated with alcohol, and sealed with neutral resin. After the sections were air‐dried, the tissue immunohistochemistry images were observed under a microscope.

Following the same steps as in the last step for deparaffinization, dehydration, and antigen retrieval of mouse Hepa1‐6 tumor tissue sections, a PBS solution containing 0.3% Triton‐X‐100 was added to the sections for membrane permeabilization for 30 min, followed by washing the sections with PBS. Subsequently, primary and secondary antibody solutions were added, and the tissues were washed as described above; 10 µg/mL DAPI solution was dripped onto the sections in the dark, and the tissues were incubated at room temperature for 10 min. After washing with PBS, an anti‐fade mounting medium was used to seal the sections, which were allowed to stand at room temperature for 24 h until fully fixed. The sections were then observed under a fluorescence microscope.

### RNA extraction and PCR

The tissues with 1 mL TRizol were transferred to a 1.5 mL RNase‐free EP tube, inverted 10–15 times, and placed on ice for 5 min. The tube was centrifuged at 12,000 × *g*, 4°C, for 5 min. After centrifugation, the supernatant (1000 μL) was transferred to a new tube. Then, 200 μL chloroform was added to each tube in a TRizol:chloroform ratio of 5:1 in the dark, inverted 10–15 times, incubated on ice for 5 min, and centrifuged at 4°C, 10,000 × *g* for 15 min. The supernatant (400–500 μL) was transferred to a new EP tube. An equal volume of isopropanol was added, and the tube was inverted a few times, incubated on ice for 10 min, and centrifuged at 4°C, 10,000 × *g* for 10 min until a visible white flocculent precipitate appeared. The supernatant was discarded, and 1 mL of 75% ethanol was added. The precipitate was resuspended, inverted evenly, and centrifuged at 4°C, 10,000 × *g* for 5 min. The supernatant was removed and air‐dried on a clean bench for 10 min, after which RNA‐free H_2_O (30 μL for minimal precipitate, 100–300 μL for abundant precipitate) was added and gently resuspended. A reverse transcription kit was used to reverse transcribe 1 µg RNA for subsequent experiments, and the reverse‐transcribed product was stored at −20°C.

### Western blot experiment

Human LM3 and SK‐HEP1 cells were cultured in the logarithmic growth phase. The cells were washed twice with PBS, and 1% RIPA lysis buffer containing protease and phosphatase inhibitors was added. The cells were lysed on ice for 10 min, centrifuged at 12,000 × *g* for 10 min at 4°C, and the supernatant was collected to obtain the total protein extract. The standards and samples were added to a 96‐well plate, BCA working solution was added, and the plate was incubated at 37°C for 30 min. The absorbance was measured at 562 nm using a microplate reader. The protein concentration was calculated based on a standard curve. Equal amounts of protein were mixed with 5 × SDS loading buffer, denatured at 95°C for 5 min, and separated using SDS‐PAGE. After electrophoresis, the proteins were transferred onto polyvinylidene fluoride membranes and blocked with 5% nonfat milk in TBST at room temperature for 90 min. A diluted primary antibody was added and incubated overnight at 4°C. The samples were washed three times with TBST for 10 min each. Horseradish peroxidase‐conjugated secondary antibodies were added, incubated at room temperature for 90 min, and washed three times with TBST for 10 min each. In a dark room, the ECL chemiluminescent reagent was evenly applied to the membrane, and band images were captured using a chemiluminescence imaging system. The experiments were repeated at least three times to ensure reproducibility. The data were analyzed using ImageJ software to calculate the grayscale ratio of the target protein to the internal reference protein.

### CCK‐8

When human (HCC‐LM3 and SK‐HEP1) and mouse HCC cells (Hepa1‐6) reached 80–85% confluence, they were digested and harvested using a 0.25% trypsin‐EDTA solution. The cells were seeded in 96‐well plates at a density of 5 × 10^3^ cells per well in 100 μL complete DMEM containing 10% FBS and 1% penicillin–streptomycin. The plates were incubated in a humidified incubator at 37°C with 5% CO_2_ for 24 h to allow cell attachment. The media were replaced with fresh media containing PBS (control group), free SPIO, sgRRM2, or SPIO‐sgRRM2. After 24 h of treatment, 10 μL of CCK‐8 solution (Dojindo Laboratories) was added to each well and incubated under standard conditions in the dark for 1.5 h. Absorbance was measured using a microplate reader at 450 nm. Three replicate wells were set up for each treatment condition, and three independent experiments were performed to ensure reproducibility. Data were analyzed using GraphPad Prism software, and cell viability was calculated (treatment group absorbance/control group absorbance × 100%) during the experiment to ensure uniform cell seeding density, avoid well‐to‐well variation, and regularly monitor the CO_2_ concentration and humidity in the incubator to maintain stable culture conditions.

### MDA, GSH, and iron ion detection

HCC‐LM3 and SK‐HEP1 cells were seeded in 6‐well plates at a density of 2 × 10^5^ cells/well and cultured in complete DMEM until reaching 70%–80% confluence. Cells were then treated with Erastin (10 μmol/L, Sigma‐Aldrich) or vehicle control (DMSO, final concentration 0.1%) for 24 h under standard culture conditions.

Following treatment, the cells were harvested using trypsin‐ethylenediaminetetraacetic acid, washed twice with cold PBS, and counted using a hemocytometer. The cell pellets were resuspended in ice‐cold lysis buffer (provided in the respective assay kits) at a concentration of 1 × 10^6^ cells/mL. The samples were sonicated using a probe sonicator and stored on ice. The lysates were centrifuged at 8,000 × *g* for 10 min at 4°C, and the supernatants were collected and stored at 4°C for immediate analysis.

Lipid peroxidation was assessed using an MDA assay kit. Then, 200 μL of supernatant was mixed with 600 μL TBA solution and incubated at 95°C for 60 min. The absorbance was measured at 532 nm. GSH levels were determined using a GSH Assay Kit. The supernatant (50 μL) was mixed with 150 μL of the freshly prepared assay cocktail in a 96‐well plate. The absorbance was measured at 412 nm after 25 min of incubation at room temperature. Iron content was measured using an Iron Assay Kit. The supernatant (50 μL) was mixed with a 50 μL iron reducer and incubated for 30 min, followed by the addition of 200 μL of iron probe. The absorbance was measured at 593 nm after 60 min of incubation at room temperature.

All measurements were performed using a microplate reader. Standard curves were generated using known concentrations of MDA, GSH, and iron standards provided in the respective kits. The results were normalized to the total protein content, which was determined using the BCA protein assay. Each experiment was performed in triplicate and repeated three times independently.

### Transmission electron microscopy

Human LM3 and SK‐HEP1 cells in the logarithmic growth phase were harvested and washed 2–3 times with pre‐warmed PBS (37°C). Then, 0.25% trypsin was added and digested at 37°C for 3–5 min, and the cells were collected through centrifugation at 1000 × *g* for 3 min. After washing once with PBS, cells were fixed with 2.5% glutaraldehyde (in 0.1 M phosphate buffer, pH 7.4) at room temperature for 2 h, followed by centrifugation at 1000 × g for 3 min to discard the supernatant. The cell pellet was resuspended in a pre‐warmed 1% agarose solution (40–45°C). The samples were fixed in 2.5% glutaraldehyde for 2 h and dehydrated using an ethanol gradient. Isoamyl acetate was replaced twice (15 min each), followed by infiltration with Epon 812 (at ratios of 2:1, 1:1, and 1:2 with isoamyl acetate for 1 h each) and pure Epon 812 overnight. Polymerized at 60°C for 48 h, ultrathin sections (60–80 nm) were prepared and stained with uranyl acetate (15 min) and lead citrate (10 min). Finally, the specimens were observed and photographed using a transmission electron microscope, and proper scale bars were included in all images. All reagents were freshly prepared, pH‐adjusted, and maintained at appropriate temperatures throughout the procedure.

### Flow cytometry

HCC‐LM3 or SK‐HEP1 cells were harvested during the logarithmic growth phase, washed with PBS, and digested with 0.25% trypsin‐EDTA for 2–5 min. The cells were then resuspended in cold FACS buffer (PBS containing 2% FBS and 2 mM EDTA). Tissue samples were cut into uniform small pieces and incubated in a digestion buffer containing collagenase IV (1 mg/mL) and DNase I (100 μg/mL) at 37°C for 45–60 min. The cell concentration was adjusted to 1 × 10^6^ cells/mL. For surface staining, a dead cell exclusion dye and optimized concentrations of antibodies were added, incubated at 4°C in the dark for 30 min, and washed. For intracellular staining, cells were fixed with 4% paraformaldehyde for 15 min, permeabilized with 0.1% Triton X‐100 for 10 min, and incubated with antibodies at 4°C in the dark for 45 min before washing. Data were acquired using a flow cytometer with at least 30,000 live cells per sample. This experiment was repeated three times. Data were analyzed using FlowJo software, and the percentage of positive cells, mean fluorescence intensity, and group differences were calculated using analysis of variance (ANOVA) or *t*‐test; *p* < 0.05 was considered statistically significant.

### RNA sequencing and KEGG analysis

After washing LM3 cells with PBS, RNA was extracted from LM3 cells, and the RNA integrity was assessed, followed by RNA sequencing. First, the raw data were processed, and hisat2 was used to obtain the gene expression sequences, identify sample differential genes using the DEseq R function, and perform KEGG enrichment analysis using R software (version 4.2.0, R Foundation for Statistical Computing).

### Statistical analysis

All data generated in this study were processed using the statistical software SPSS version 21.0 (IBM Corp.). The results of comparisons between two groups of functional experimental data are expressed as the mean ± standard deviation. Differential analyses were performed using unpaired *t*‐tests, ANOVA, and two‐way ANOVA. For multiple group comparisons following ANOVA, Tukey's HSD post hoc test was applied to control for family‐wise error rate. For select analyses, Bonferroni correction was implemented to adjust significance levels based on the number of comparisons. Statistical significance was set at *p* < 0.05. We employed the Kruskal–Wallis non‐parametric test for overall between‐group differences, followed by Dunn's multiple comparison test for pairwise comparisons between groups. In the figure, the notations “NS,” and “*” represent no significant difference (*p* > 0.05), significant difference (*p* < 0.01), and highly significant difference (*p* < 0.001).

## AUTHOR CONTRIBUTIONS

**Weiliang Hou**: Writing—original draft; methodology; software; validation; data curation; conceptualization. **Weifeng Hong**: Writing—original draft; methodology; software; validation; data curation; conceptualization. **Songhua Cai**: Conceptualization; methodology; validation; software; data curation; writing—original draft. **Dandan Guo**: Writing—original draft; methodology; software; validation; data curation; conceptualization. **Zhiping Yan**: Writing—review and editing; conceptualization; methodology; data curation. **Jinyu Zhu**: Visualization; data curation; writing—review and editing; software. **Yang Shen**: Writing—review and editing; data curation; software; validation. **Juncheng Wan**: Data curation; writing—review and editing; validation; software. **Xudong Qu**: Software; data curation; validation; writing—review and editing. **Wen Zhang**: Software; supervision; validation; writing—review and editing. **Runkang Zhao**: Software; investigation; writing—review and editing. **Zhao Xie**: Visualization; writing—review and editing; software. **Zhongji Chen**: Data curation; investigation; writing—review and editing. **Tong Jiang**: Visualization; investigation; writing—review and editing. **Yaling Lin**: Data curation; investigation; writing—review and editing. **Wenlong Jia**: Conceptualization; writing—review and editing; data curation. **Ling Wang**: Conceptualization; writing—review and editing; data curation. **Zhao Huang**: Writing—review and editing; data curation; project administration; supervision; formal analysis. **Xuexin Li**: Validation; formal analysis; supervision; writing—review and editing; project administration. **Bufu Tang**: Writing—review and editing; funding acquisition; methodology; visualization; software; data curation; supervision.

## CONFLICT OF INTEREST STATEMENT

The authors declare no conflicts of interest.

## ETHICS STATEMENT

The ethics application (No. NCC2025A010) was approved by the Research Ethics Committee of the Institute of the Cancer Hospital Chinese Academy of Medical Sciences.

## Supporting information


**Figure S1:** Quality control and batch effect correction of scRNA‐seq data.
**Figure S2:** Ribonucleotide reductase M2 (*RRM2*) is closely associated with poor prognosis in hepatocellular carcinoma (HCC).
**Figure S3:** Time‐dependent cellular uptake dynamics and transgene expression efficiency of nanodelivery systems in HCC.
**Figure S4:** SPIO and *sgRRM2* co‐delivery nanocarriers promote ferroptosis in HCC.
**Figure S5:** *RRM2* knockout potentiates RFA‐induced immunogenic cell death in HCC.
**Figure S6:** Effects of RFA combined with different treatments on histopathological changes, biochemical parameters, and inflammatory cytokine expression in mice.
**Figure S7:** Dual‐loaded nanoparticle co‐delivery system inhibits HCC progression by inducing ferroptosis.

## Data Availability

The data that support the findings of this study are available on request from the corresponding author. The data are not publicly available due to privacy or ethical restrictions. All data are publicly available for download. The Cancer Genome Atlas Program (TCGA) data are available at https://www.cancer.gov/ccg/research/genome-sequencing/tcga. The Gene Expression Omnibus (GEO) data are available at: https://www.ncbi.nlm.nih.gov/geo/query/acc.cgi?acc=GSE242889; https://www.ncbi.nlm.nih.gov/geo/query/acc.cgi?acc=GSE149614; https://www.ncbi.nlm.nih.gov/geo/query/acc.cgi?acc=GSE43036; https://www.ncbi.nlm.nih.gov/bioproject/PRJNA1280041. In this study, the RNA sequencing data for the self‐tested HCC‐LM3 cells are available at https://biosys.bgi.com/#/report/reanalysis/index, and https://biosys.bgi.com/#/report/reanalysis/index. The data and scripts used are saved in GitHub https://github.com/TurboF612/HCC_Nano. Supplementary materials (figures, graphical abstract, slides, videos, Chinese translated version, and update materials) may be found in the online DOI or iMeta Science http://www.imeta.science/.
