# Supplementary material for: RRM2‐targeted nanocarrier enhances radiofrequency ablation efficacy in hepatocellular carcinoma through ferroptosis amplification and immune remodeling
Source: Imeta. 2025 Aug 6;4(5):e70067. doi: 10.1002/imt2.70067 (PMC12527986; doi:10.1002/imt2.70067)
Supplement: Supplementary file 1 — Figure S1: Quality control and batch effect correction of scRNA‐seq data. Figure S2: Ribonucleotide reductase M2 (RRM2) is closely associated with poor prognosis in hepatocellular carcinoma (HCC). Figure S3: Time‐dependent cellular uptake dynamics and transgene expression efficiency of nanodelivery systems in HCC. Figure S4: SPIO and sgRRM2 co‐delivery nanocarriers promote ferroptosis in HCC. Figure S5: RRM2 knockout potentiates RFA‐induced immunogenic cell death in HCC. Figure S6: Effects of RFA combined with different treatments on histopathological changes, biochemical parameters, and inflammatory cytokine expression in mice. Figure S7: Dual‐loaded nanoparticle co‐delivery system inhibits HCC progression by inducing ferroptosis. [file IMT2-4-e70067-s001.docx]

**Supporting information to**

**RRM2-targeted nanocarrier enhances radiofrequency ablation efficacy in hepatocellular carcinoma through ferroptosis amplification and immune remodeling**

**Running title:** RRM2-targeted nanocarrier amplifies RFA efficacy via ferroptosis in HCC

Weiliang Hou^1#^, Weifeng Hong^2#^, Songhua Cai^3#^, Dandan Guo^4#^, Zhiping Yan^5^, Jinyu Zhu^6^, Yang Shen^7^, Juncheng Wan^5^, Xudong Qu^5^, Wen Zhang^5^, Runkang Zhao^4^, Zhao Xie^4^, Zhongji Chen^4^, Tong Jiang^4^, Yaling Lin^4^, Wenlong Jia^8^, Ling Wang^4^, Zhao Huang^8*^, Xuexin Li^9,10*^, Bufu Tang^5*^

^1^Department of Gastroenterology, Shanghai Institute of Pancreatic Diseases, National Key Laboratory of Immunity and Inflammation, Changhai Clinical Research Unit, Changhai Hospital, Naval Medical University, Shanghai 200433, China.

^2^Department of Radiation Oncology, Zhejiang Cancer Hospital; Hangzhou Institute of Medicine (HIM), Chinese Academy of Sciences, Hangzhou 310022, China

^3^Department of Thoracic Surgery, National Cancer Center/National Clinical Research Center for Cancer/Cancer Hospital & Shenzhen Hospital, Chinese Academy of Medical Sciences and Peking Union Medical College, Shenzhen 518116, China

^4^First Affiliated Hospital, Dalian Medical University, Dalian 116011, China

^5^Department of Interventional Radiology, Zhongshan hospital, Shanghai Institute of Medical Imaging,Shanghai Institution of Medical Imaging, Shanghai, National Clinical Research Center of Interventional Medicine, Fudan University, Shanghai 200032, China

^6^Key Laboratory of Carcinogenesis and Translational Research (Ministry of Education/Beijing), Department of Nuclear Medicine, Peking University Cancer Hospital & Institute, Peking University, Beijing 100142, China

^7^Department of Radiation Oncology, Zhongshan Hospital Affiliated to Fudan University, Shanghai 200032, China

^8^Hepatic Surgery Center, Tongji Hospital, Tongji Medical College, Huazhong University of Science and Technology, Wuhan 430030, China

^9^Department of General Surgery, The Fourth Affiliated Hospital, China Medical University, Shenyang 110032, China

^10^Department of Physiology and Pharmacology, Karolinska Institute, Solna 171 65, Sweden

^#^These authors contributed equally: Weiliang Hou, Weifeng Hong, Songhua Cai, Dandan Guo

*Correspondence: tangbufu@zju.edu.cn (Bufu Tang), xuexin.li@ki.se (Xuexin Li), huangzhao@tjh.tjmu.edu.cn (Zhao Huang)


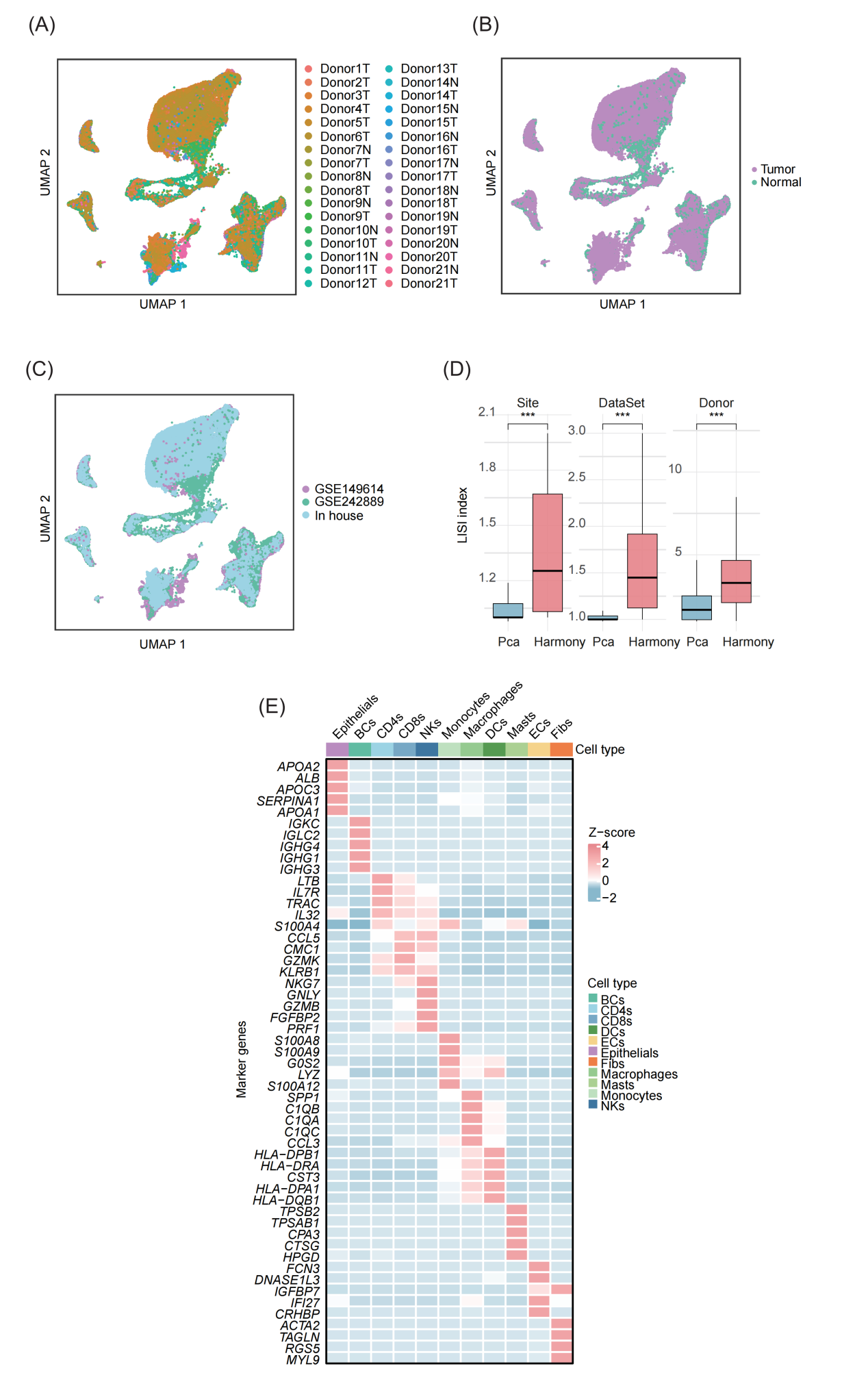


**Figure S1 Quality control and batch effect correction of scRNA-seq data.** (A) Uniform Manifold Approximation and Projection (UMAP) visualization. Distribution of cells in the UMAP reduced-dimensional space, with each color representing a different donor (donor). The distribution patterns of cells from different donors in the UMAP space can be intuitively observed through color differentiation, enabling the assessment of similarities and differences between donors and cell distribution characteristics. (B) Pie chart comparing cell compositions of normal and tumor tissues. Different colors for each cell type indicate the differences in cell proportions between normal and tumor tissues. (C) UMAP dimensionality reduction results of the scRNA-seq data after batch effect correction using both the principal component analysis (PCA) and Harmony methods. The effectiveness of batch-effect correction can be evaluated by comparing the cell distributions generated by the two methods. The Harmony method performed better than PCA at reducing batch effects, leading to a more uniform and clear cell distribution. (D) LISI boxplot comparing the LISI values of the PCA and Harmony methods across different datasets and donors. The LISI index was used to quantify the effectiveness of the batch effect correction, with higher values indicating smaller batch effects. The boxplot displays the median, quartiles, and range of the LISI index values, demonstrating that the Harmony method has significantly higher LISI values than PCA, proving its superiority in batch effect correction. (E) Displays a heatmap of characteristic marker gene expression across different cell types (epithelial cells, B cells, T cell subsets, NK cells, etc.).


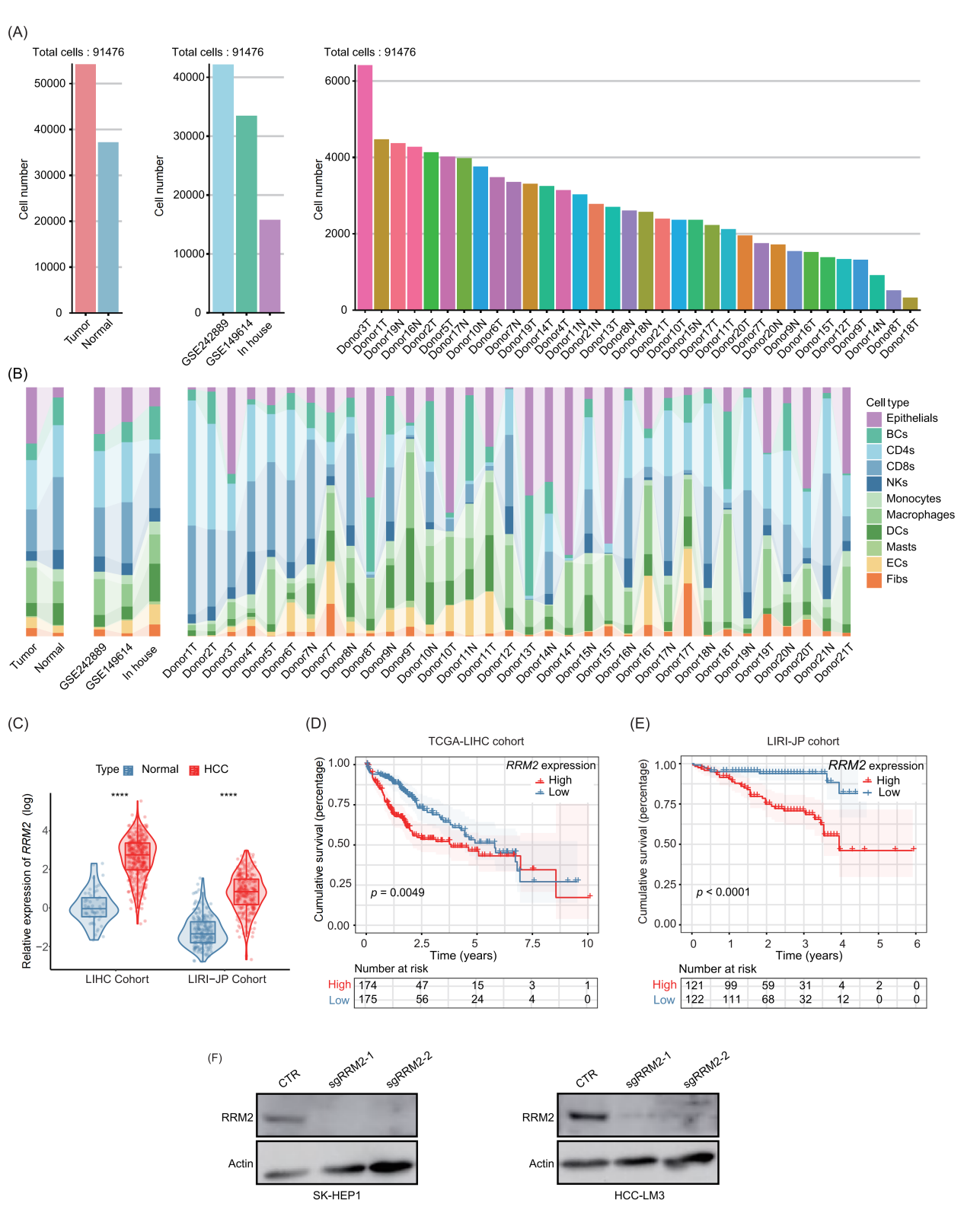


**Figure S2 Ribonucleotide reductase M2 (*RRM2*) is closely associated with poor prognosis in hepatocellular carcinoma (HCC).** (A) Cell counts from different sample sources, including tissue types, datasets, and donors, after quality control and filtering. (B) Detailed comparison of cellular composition across different tissue types, datasets, and donors. (C) *RRM2* expression in TCGA-LIHC and LIRI-JP cohorts. (D, E) Survival of patients with HCC with different *RRM2* expression levels in the TCGA-LIHC (D) and LIRI-JP (E) cohorts. (F) Western blot results showing RRM2 and actin expression in SK-HEP1 and HCC-LM3 cells treated with control, sgRRM2-1, and sgRRM2-2.


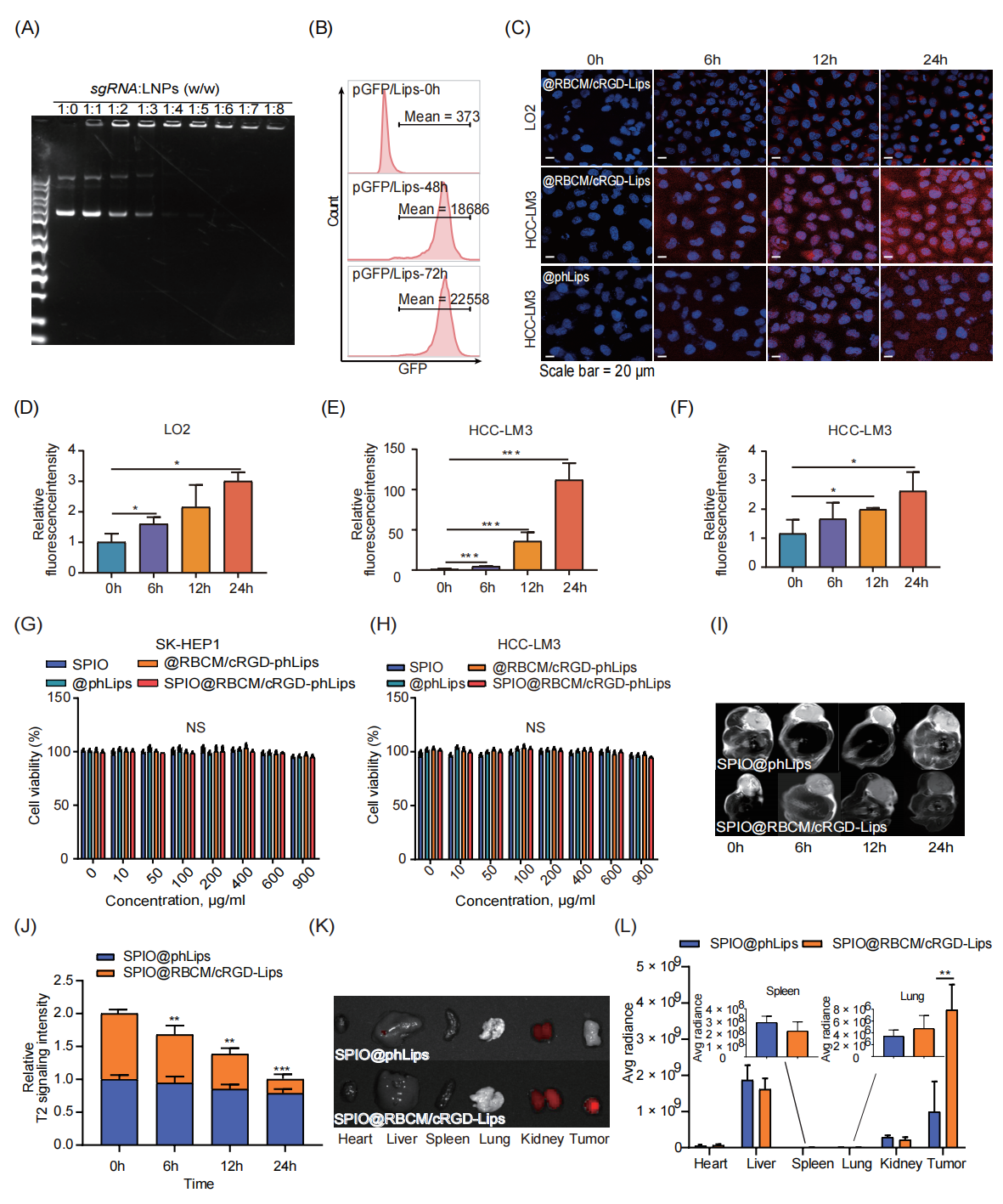


**Figure S3 Time-dependent cellular uptake dynamics and transgene expression efficiency of nanodelivery systems in HCC.** (A) Agarose gel electrophoresis showing successful formation and stability of LNP-sgRNA complexes at varying mass ratios. (B, C) Representative immunofluorescence (IF) images of LO2 and HCC-LM3 cells, demonstrating specific and enhanced uptake of lipid nanoparticles (LNPs) by HCC-LM3 cells. (D-F) Time-course analysis of mean fluorescence intensity (MFI) in LO2 and HCC-LM3 cells, showing preferential accumulation of LNPs in HCC-LM3 cells. (G, H) Viability assays of SK-HEP-1 (G) and HCC-LM3 (H) cells treated with different LNP formulations. (I) Representative magnetic resonance imaging scans of HCC tumors under various treatment conditions with SPIO@RBCM/cRGD-phLips showing enhanced tumor contrast. (J) Quantitative analysis of the relative T2-weighted MRI signal intensity, indicating superior tumor imaging with SPIO@RBCM/cRGD-phLips. (K) In vivo fluorescence imaging demonstrating Cy5.5 accumulation in various organs after the administration of @phLips or @RBCM/cRGD-phLips. (L) Quantitative analysis of bioluminescence intensity in various organs, confirming higher tumor-specific accumulation of SPIO@RBCM/cRGD-phLips than SPIO@phLips. Scale bar = 20 μm.*, *p* < 0.05; **, *p* < 0.01; ***, *p* < 0.001.


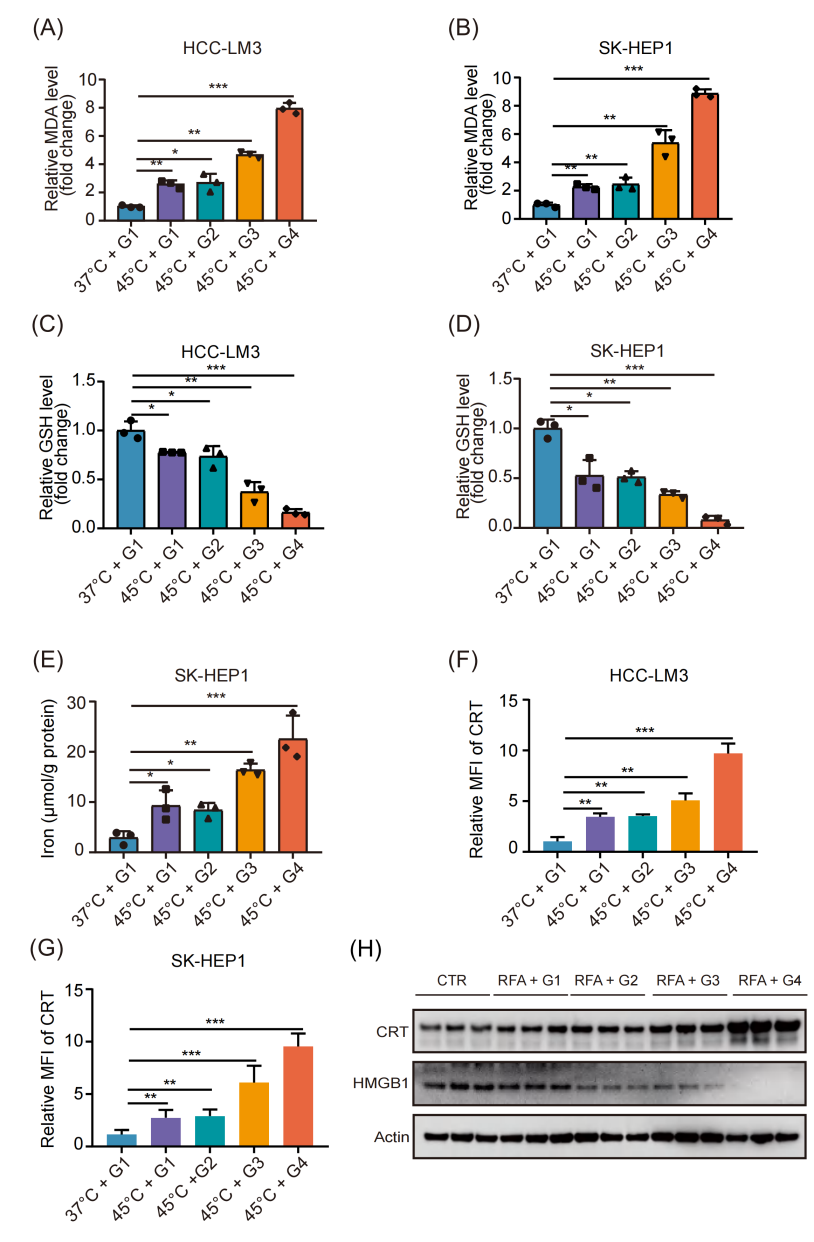


**Figure S4 SPIO and *sgRRM2* co-delivery nanocarriers promote ferroptosis in HCC.** (A, B) Malondialdehyde (MDA) content in HCC-LM3 (A) and SK-HEP1 (B) cells under different treatment conditions. (C, D) Glutathione (GSH) content in HCC-LM3(C) and SK-HEP1(D) cells under different treatment conditions. (E) Iron content in SK-HEP1 cells under different treatment conditions. (F, G) Relative MFI intensity of HCC-LM3 (F) and SK-HEP1 cells (G) under different treatment conditions. (H) Expression profiles of calreticulin (CRT) and high-mobility group box 1 (HMGB1) under radiofrequency ablation and various nanoparticle treatment conditions. *, *p* < 0.05; **, *p* < 0.01; ***, *p* < 0.001.


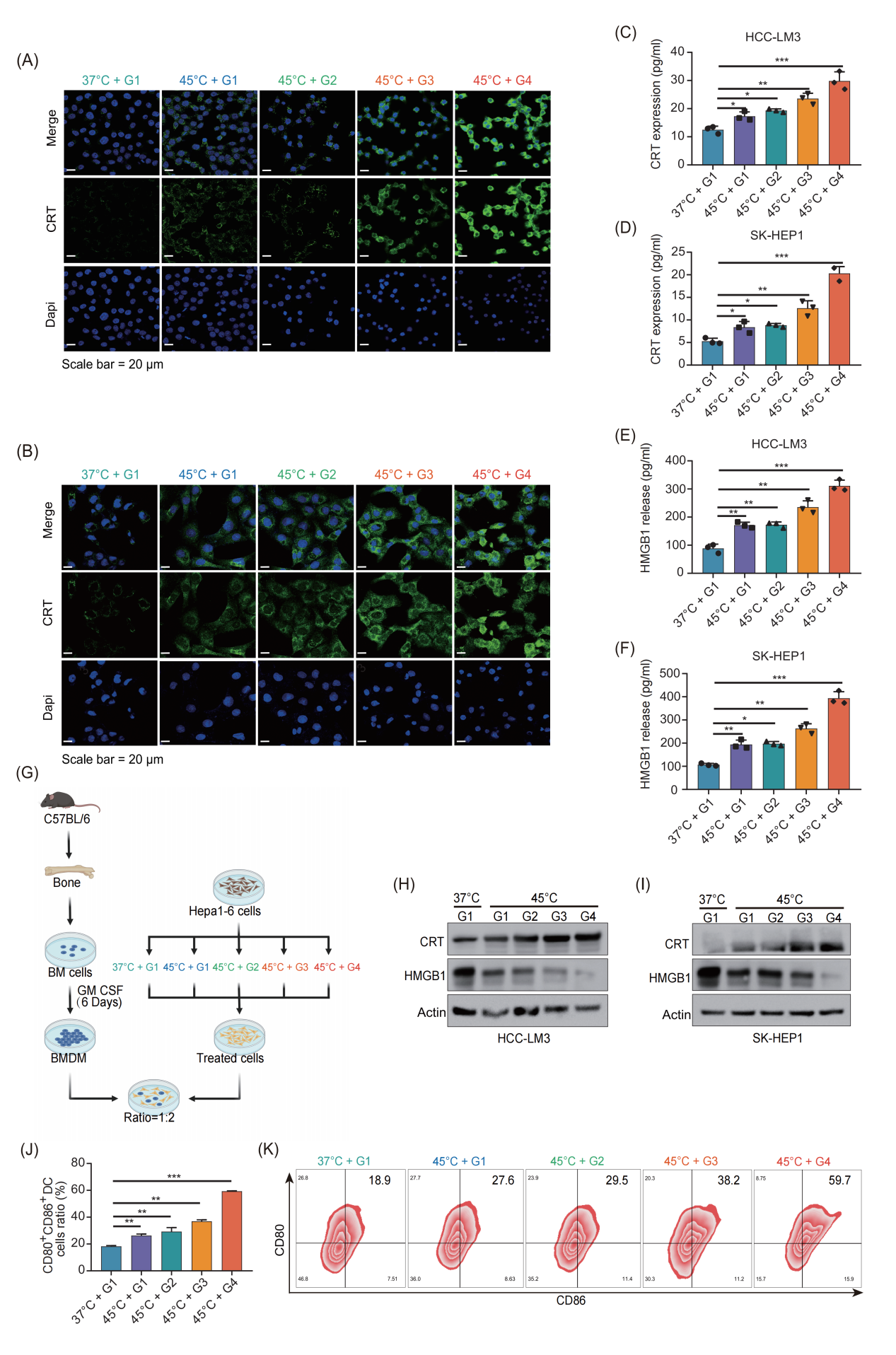


**Figure S5 *RRM2* knockout potentiates RFA-induced immunogenic cell death in HCC.**

(A, B) Representative confocal laser scanning microscopy images showing increased surface expression of calreticulin (CRT), a hallmark of ICD, under RFA treatment, which is further amplified by *RRM2* knockout. Scale bars = 10 µm. (C, D) Quantitative analysis of CRT expression levels in HCC-LM3 (C) and SK-HEP1 (D) cells under different treatment conditions, demonstrating significantly higher CRT expression in the RFA + *RRM2* knockout group compared to RFA alone. (E, F) Quantification of high-mobility group box 1 (HMGB1) release from HCC-LM3 (E) and SK-HEP1 (F) cells, showing the enhanced extracellular release of HMGB1, another ICD marker, under combined RFA and *RRM2* knockout treatment. (G) Schematic diagram illustrating the experimental workflow, including cell culture, RFA treatment, and *RRM2* knockout interventions. (H, I) Western blot analysis of CRT and HMGB1 protein expression in HCC-LM3 (H) and SK-HEP1 (I) cells, with actin as a loading control, confirming increased ICD-related marker expression in the RFA + *RRM2* knockout group. (J) Quantitative analysis of CD80+ and CD86+ cell percentages, indicating enhanced activation of antigen-presenting cells after RFA + *RRM2* knockout treatment. (K) Representative flow cytometry pseudocolor plots showing elevated CD80 and CD86 expression in live cells isolated from HCC tumor tissues treated with RFA + *RRM2* knockout, suggesting enhanced immune activation. Scale bar = 20 μm. *, *p* < 0.05; **, *p* < 0.01; ***, *p* < 0.001.


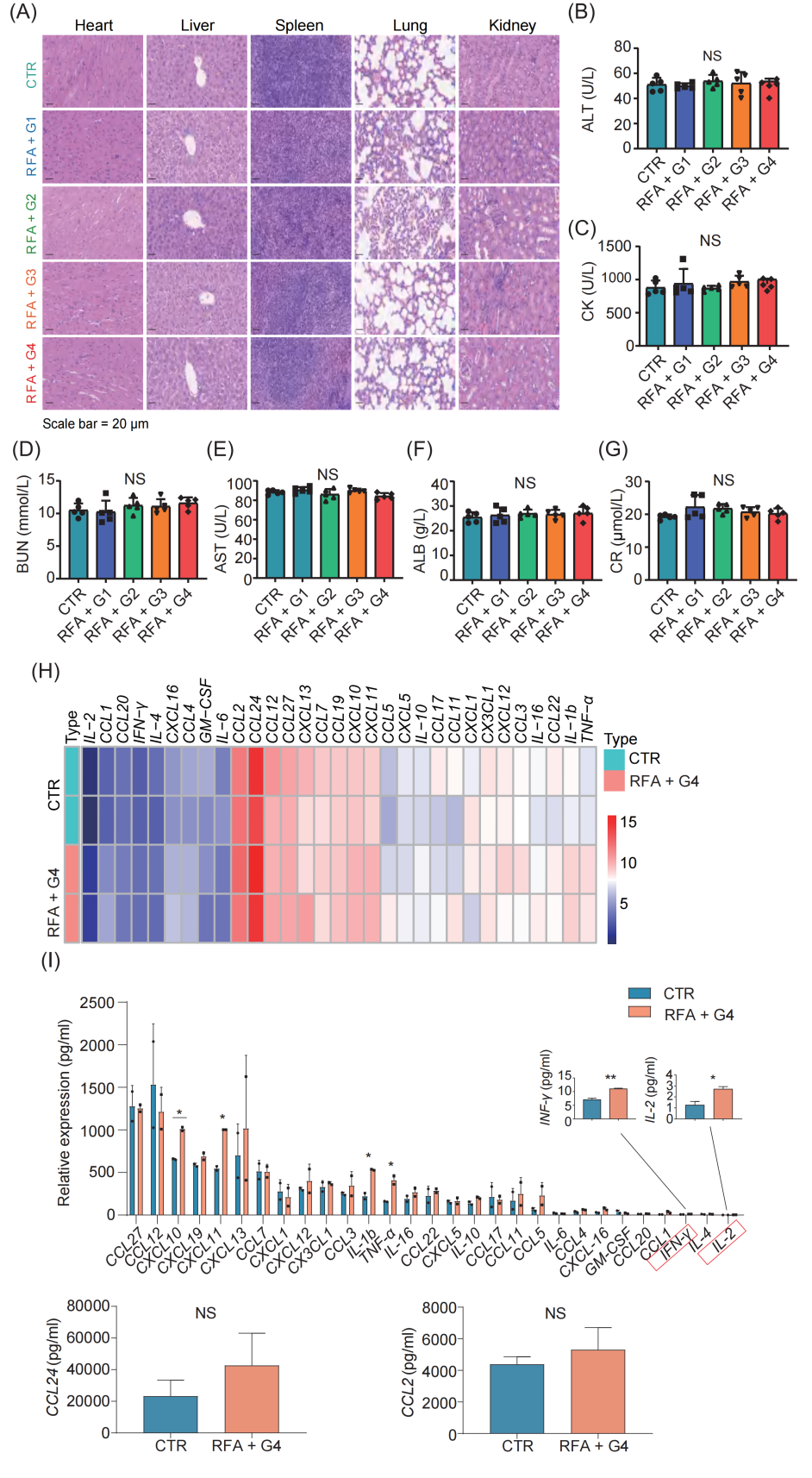


**Figure S6 Effects of RFA combined with different treatments on histopathological changes, biochemical parameters, and inflammatory cytokine expression in mice.** (A) Representative hematoxylin and eosin images show the structures of the heart, liver, spleen, lungs, and kidneys after treatment in each group of mice. (B-G) Alanine aminotransferase (ALT), creatine kinase (CK), blood urea nitrogen (BUN), aspartate aminotransferase (AST), albumin (ALB), and creatinine (CR) levels in each mouse group after drug treatment. (H) Multiple cytokine arrays show cytokines secreted by HCC. (I) Cytokine secretion statistics and the levels of C-C motif chemokine ligand 24 (*CCL24*) and *CCL2* detected by multiple cytokine assay.


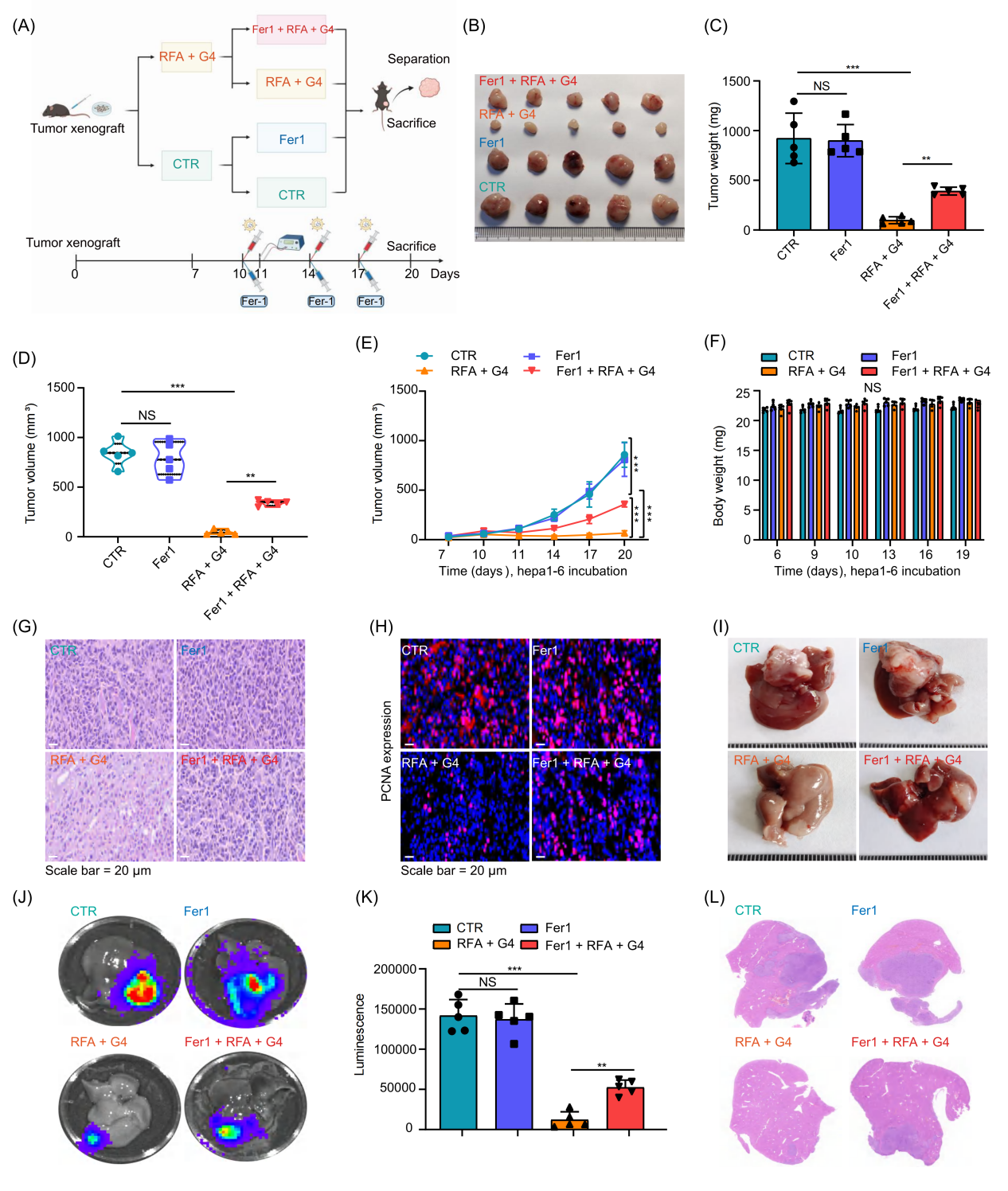


**Figure S7 Dual-loaded nanoparticle co-delivery system inhibits HCC progression by inducing ferroptosis.** (A) Flowchart of the experimental design for an animal model of HCC treated with the nanoparticle codelivery system, ferroptosis inhibitor (Fer-1), and RFA, outlining the treatment timeline and interventions. (B) Tumor size comparisons under different treatment conditions, showing significant tumor suppression in the RFA + nanoparticle group compared with the other treatments (*n* = 5). (C) Histogram of tumor weight statistics across groups, with the lowest tumor weight observed in the RFA + nanoparticle group. (D) Statistical violin plot of tumor volume demonstrating reduced tumor size variability and smaller tumor volumes in the RFA + nanoparticle group. (E) Line graph showing tumor growth kinetics, with markedly slower tumor growth in the RFA + nanoparticles group than in the control or Fer-1-treated groups. (F) Statistical histogram of changes in mouse weight, indicating minimal systemic toxicity and stable body weight in all treatment groups. (G) Representative H&E staining of tumor tissues under different treatments showing extensive tumor necrosis and structural damage in the RFA + nanoparticle group. (H) Immunohistochemical analysis of PCNA expression (a proliferation marker) under various treatments with a significant reduction in PCNA levels in the RFA + nanoparticle group, indicating suppressed tumor cell proliferation. (I) Representative images of in situ tumor models demonstrating smaller tumors in the RFA + nanoparticle group. (J, K) IVIS imaging of in situ tumor models with quantitative analysis of luminescence intensity revealed significantly reduced tumor signals in the RFA + nanoparticle group. (L) Representative H&E-stained images confirming extensive ferroptosis-induced damage in tumor tissues treated with the co-delivery system. Significant iron particle deposition was observed within the liver tissue, indicating that ferroptosis has led to substantial iron accumulation. Scale bar = 20 μm. *, *p* < 0.05; **, *p* < 0.01; ***, *p* < 0.001.
